# Efficacy and Safety of Korean Herbal Medicine for Patients with Post-Accident Syndrome, Persistent after Acute Phase: A Pragmatic Randomized Controlled Trial

**DOI:** 10.3390/healthcare11040534

**Published:** 2023-02-10

**Authors:** Bo-Kyung Hwang, Kyoung Sun Park, Seung-Hyeok Ku, Sung-Hyun Kim, Hyun-Woo Moon, Mi-So Park, Hye-Kyung Baek, Jin Namgoong, Seung-Yoon Hwangbo, Ji-Yeon Seo, Yoon Jae Lee, Jinho Lee, In-Hyuk Ha

**Affiliations:** 1 Bucheon Jaseng Hospital of Korean Medicine, 17, Buil-ro, 191beon-gil, Bucheon-si 14598, Republic of Korea; 2 Jaseng Hospital of Korean Medicine, 536, Gangna-daero, Gangnam-gu, Seoul 06110, Republic of Korea; 3 Jaseng Spine and Joint Research Institute, Jaseng Medical Foundation, 2F 540 Gangnam-daero, Gangnam-gu, Seoul 06110, Republic of Korea

**Keywords:** road traffic accident, post-accident syndrome, whiplash-associated disorder, herbal medicine, impact of event scale-revised-Korean

## Abstract

This is a pragmatic, two-armed, parallel, single-center, randomized controlled clinical trial for comparative evaluation between the effectiveness of integrated Korean medicine (IKM) and herbal medicine treatment with that of IKM monotherapy (control) for post-accident syndrome persistent after the acute phase. Participants were randomized into Herbal Medicine (HM, *n* = 20) and Control groups (*n* = 20) to receive the allocated treatment of 1–3 sessions/week for 4 weeks. Intention-to-treat analysis was conducted. The Difference of Numeric Rating Scale (NRS) change of overall post-accident syndromes from baseline to week 5 for the two groups was 1.78 (95% CI: 1.08–2.48; *p* < 0.001). Regarding secondary outcomes, a significant decrease compared to the baseline values was confirmed for NRS of musculoskeletal, neurological, psychiatric complaints and general symptoms of post-accident syndromes. In a survival analysis based on the recovery criteria of “patients with a reduction in the NRS of overall post-accident syndromes of ≥50%,” the HM group showed a shorter time to recovery than the control group during the 17-week study period (*p* < 0.001 by the log-rank test). IKM combined with herbal medicine treatment significantly improved the quality of life by relieving somatic pain and alleviating the overall post-accident syndrome persistent after the acute phase; this effect was maintained for at least 17 weeks.

## 1. Introduction

Automobiles are an essential means of transportation in modern human civilization, and over 50% of households in major developed countries have automobiles [1]. An increasing number of automobiles and escalating complexity in logistics and volume of traffic, in line with rapid economic growth, has led to the unfortunate consequence of an increasing rate of road traffic accidents. More than 3 million accidents occur worldwide every year in the 30 Organisation for Economic Co-operation and Development countries [2], which translates into a large number of patients with road traffic injuries.

Patients with road traffic injuries complain of various sequelae and injuries to the musculoskeletal system, such as the neck, lower back, and shoulder, caused upon impact at the time of the accident. In particular, whiplash injuries, caused by a forceful, sudden hyperextension followed by hyperflexion of the cervical vertebrae due to abrupt acceleration or deceleration, are fairly common. Whiplash injury is known to cause symptoms, such as headache, dizziness, fatigue, and insomnia, in addition to pain caused by soft tissue injury around the neck and shoulder [3]. In addition, post-accident syndrome is characterized by the difficulty in achieving a full recovery with short-term treatment alone. Previous studies reported that only one-third of the patients recovered from the initial pain and disability within 3 months after a whiplash injury, and two-thirds of the patients complained of persistent pain even after 3 months [4]. Even after 6 months after the accident, patients complained of symptoms such as palpitations, gastrointestinal symptoms, shortness of breath, and sleep disturbances, as well as headache and neck pain [5]. Furthermore, chronic headaches, pain in the neck and lower back, and chronic fatigue persist for several years [6]. As understood from the literature, many patients suffer from somatic pain and other symptoms even after the acute phase has elapsed, and therefore, post-accident syndrome is said to be characterized by the persistence of various symptoms and a longer time for complete recovery.

To date, the guidelines for Western medicine treatment regarding whiplash-associated disorder recommend conservative treatments to relieve pain through injections and drugs in general, although the level of evidence is not high. With the Western medicine approach, trigger point treatment is administered for treating myofascial components through the injection of local anesthetics, sterile water, botulinum toxin, steroids, etc., or prescribing medications, such as non-steroidal anti-inflammatory drugs (NSAIDs). For symptoms such as hyperalgesia and sleep disorder associated with pain or depression after an accident, antidepressants or anticonvulsants may be used, or opioids may be prescribed with extreme caution [7]. Physical therapy, rehabilitation, exercise programs, and counseling and feedback training with patients are also widely applied [8,9]. On the contrary, Korean medicine recommends pharmacoacupuncture, Chuna manual therapy, cupping, Korean medicine physiotherapy, and others, along with pharmacological treatment (herbal medicine) and acupuncture (electroacupuncture and motion-style acupuncture) for road traffic injuries [10]. In a majority of cases, patients with road traffic injuries who visit Korean medicine (KM) institutions, including KM clinics and hospitals, receive integrative Korean medicine (IKM) treatment that combines herbal medicine, acupuncture, pharmacoacupuncture, Chuna manual therapy, etc. Acupuncture has a significant therapeutic effect on neck pain in patients with whiplash-associated disorders after a traffic accident [3,11,12,13], and Chuna manual therapy has also been proven effective for 132 patients with subacute whiplash injury through a previous randomized controlled trial (RCT) [14]. In addition, IKM treatment that combines acupuncture, pharmacoacupuncture, herbal medicine, and Chuna manual therapy is effective in reducing the symptoms of patients with road traffic injuries [15]. 

As a part of the Korean herbal medicine treatment, a complex extract of medicinal herbs is prescribed by a Korean medicine doctor (KMD) who has completed specialized education and training based on the diagnosis and pattern identification of the individual’s health condition and symptoms of road traffic injuries. This treatment is one of the representative treatment modalities of Korean medicine, and it is frequently used within the automobile insurance system of South Korea. However, there are many difficulties in the standardization of Korean herbal medicine treatment because the process of pattern identification is performed according to the KMD’s judgment of each patient’s constitution. Therefore, there have been no RCTs that have demonstrated the effectiveness of herbal medicine treatment for road traffic accident syndrome. Consequently, in this study, a pragmatic, randomized controlled clinical trial was conducted to evaluate the efficacy and safety of herbal medicine treatment for patients with post-accident syndrome after the acute phase. The hypothesis of this study is that for post-accident syndrome after the acute phase, a combination of IKM treatment and herbal medicine treatment shows superior efficacy and safety in alleviating post-accident syndrome than IKM monotherapy. 

## 2. Methods

### 2.1. Study Design and Setting

A two-armed, parallel, single-center, randomized controlled study was designed, in which 40 patients were recruited at the Bucheon Jaseng Hospital of Korean Medicine in South Korea. The Korean Ministry of Health and Welfare (MOHW) has designated Bucheon Jaseng Hospital of Korean Medicine as a Spine-Specialty Korean Medicine Hospital and certified it for safe and quality patient care.

### 2.2. Participant Timeline

At the first visit, the participants were informed about the study and asked to complete the Informed Consent Form (ICF). Then, an assessor screened the participants for the study and recruited them according to the inclusion/exclusion criteria. From the second visit, i.e., the first visit in the first week after the initiation of the study (week 1-1), the participants were divided into The Herbal Medicine (HM) group and Control group through randomization and then administered treatment. The treatment sessions were held 1–3 times a week for 4 weeks. Next, the participants were followed up at 5, 9, and 17 weeks from the baseline. During the follow-up period, the participants either personally visited the hospital or telephonic surveys were conducted. The time schedule of the enrollment, interventions, and assessments for participants is shown in Supplementary Table S1.

### 2.3. Inclusion/Exclusion Criteria

Forty patients were enrolled after the screening process. The inclusion criteria for the study participants are as follows:

1. Outpatients who visited our hospital due to symptoms following road accidents from 1 July 2021 to 31 May 2022;
2. Male and female patients aged 19–70 years;
3. At the time of reporting to the hospital, more than 8 weeks but less than 24 weeks had elapsed since the patients were in a road traffic accident;
4. Patients with NRS ≥ 5 for post-accident syndromes;
5. Patients who have provided consent to participate in the trial and signed the informed consent form.

The exclusion criteria were as follows:

1. Patients whose pain was not caused by road traffic accidents but by existing underlying conditions (hernia of intervertebral discs, spinal canal stenosis, fibromyalgia, etc.);
2. Patients who had undergone surgery owing to injuries sustained in the current road traffic accident;
3. Patients with progressive neurological deficits or with severe neurological symptoms;
4. Patients with other chronic conditions, such as cardiovascular disease, kidney disease, diabetic neuropathy, dementia, or epilepsy, which could interfere with the interpretation of the therapeutic effects or results;
5. Patients who were taking corticosteroids, immunosuppressants, mental health medications, or other drugs that could have affected the outcomes of the study;
6. Patients with test results over twice the normal reference range for liver function tests and renal function tests at the time of screening;
7. Patients with kidney or liver/biliary system disease (hepatitis, fatty liver, cirrhosis, liver cancer, biliary obstruction, etc.);
8. Patients with gastrointestinal dysfunction or those who had undergone a surgery that could affect drug absorption, such as gastrectomy;
9. Patients who were pregnant, lactating, or planning to become pregnant;
10. Patients with a serious mental illness;
11. Patients who were participating in clinical trials, except observational studies without therapeutic intervention;
12. Patients who had difficulties in signing the informed consent form;
13. Other cases of patients whose participation in the trial was deemed not appropriate by an investigator.

### 2.4. Interventions

#### 2.4.1. Control Group: IKM Treatment

The Control group made outpatient visits twice a week for a total of 4 weeks after study enrollment, and at each visit, they received IKM treatment, such as acupuncture, pharmacoacupuncture, cupping, and Chuna manual therapy at the discretion of the KMD. In principle, visits were made twice a week, but depending on the symptoms of the patient or the individual circumstances of the patient, the number of visits was adjusted by ±1, such that the treatment could be performed at least once a week and up to 3 times a week.

In acupuncture treatment, the physician inserted the standardized disposable stainless steel filiform needle (0.25 mm × 0.30 mm, Dongbang Medical, Boryeong, Republic of Korea) into selected acupuncture points and tender points according to the judgment of the KMD (having clinical experience ≥3 years), at a depth of 10 mm, at 6–12 points in total. The needles were inserted according to the characteristics of each acupuncture point, followed by twisting and twirling of the needles for the Deqi sensation. Each acupuncture session was carried out for 10–15 min, including needle retention time, and the acupuncture treatment was performed during every visit of the patient. Pharmacoacupuncture was performed using a standardized disposable syringe (1 mL, 29 G × 1/2 syringe, Shinchang Medical, Sungnam, Republic of Korea). By identifying the patterns of the symptoms of the patient, Shinbaro (Jaseng herbal dispensary, Sungnam, Republic of Korea), Hwangryunhaedok, (Jaseng herbal dispensary, Sungnam, Republic of Korea), Joongseongouhyul (Jaseng herbal dispensary, Sungnam, Republic of Korea), and bee venom (Jaseng herbal dispensary, Sungnam, Republic of Korea) were administered prior to the acupuncture, and the injection was given subcutaneously at a depth of 1 cm and at an injection volume of 0.25 cc, at the pain site and related meridian points. 

Chuna manual therapy is a manually performed therapy used in Traditional Korean medicine, in which the KMD uses his/her hands, part of his/her body, or tools such as the Chuna table to stimulate the patient’s body structures to treat structural or functional problems. The treatment was performed by trained KMDs who had completed the standardized Chuna education course. The method and number of sessions were determined at the discretion of the KMD. Doin therapy is a technique that utilizes a breathing method and passive or active movements [3]. It assesses abnormal or limited movements in patients and helps to develop strength and flexibility in the muscles and tendons [16]. Doin therapy was performed manually by a KMD for patients with limited range of motion (ROM) or complaints of residual symptoms. Moxibustion and cupping were performed at the acupuncture treatment session according to the judgment of the KMD, and these treatments were performed on meridians and pain sites related to the area of complaint. All of the treatment methods used were recorded on the electronic chart, and the chart was reviewed retrospectively and recorded on the case report form (CRF) for analysis.

#### 2.4.2. Experimental Group: IKM Treatment + Herbal Medicine Treatment

The HM group made outpatient visits twice a week for a total of 4 weeks after study enrollment, and at each visit, they received IKM treatment, such as acupuncture, pharmacoacupuncture, cupping, and Chuna manual therapy, at the discretion of the KMD. In principle, visits were made twice a week, but depending on the severity of the symptoms of the patient or the individual circumstances of the participant, the number of treatments was adjusted to at least once a week and up to 3 times a week.

For herbal medicine treatment, the patients in the HM group took herbal medicine prescribed according to the identification of patterns of the patient’s symptoms by a KMD. At the first visit in the first week (Week 1-1), the first prescription for 14 days was dispensed, and at the visit Week 3-1, two weeks after the firsts visit, the second prescription for 14 days was dispensed. The type and dosing regimen of herbal medicine were not determined in advance, but rather, a KMD, i.e., a physician, determined the medicines, dosages, and regimens, according to the patient’s condition and complaints and based on his/her clinical assessment and judgment. For prescribing the second round of herbal medicine, the same or a different type of herbal medicine was prescribed according to the patient’s complaints and treatment progress. However, in the event of adverse events, such as diarrhea, abdominal pain, feeling of fullness in the abdomen, or indigestion, upon taking the prescribed herbal medicine, the prescription was changed, or an additional medicine was prescribed as an adjunct to alleviate the symptoms, based on the physician’s judgment.

To ensure accurate evaluation, all information related to the procedure and herbal medicine prescription was recorded on the electronic charts. The information on the performed treatments and prescribed herbal medicine was reviewed on the chart retrospectively and recorded on the CRF for analysis.

### 2.5. Criteria for Premature Termination and Dropout

The study was terminated early for the applicable participant in case of the following events:

(1) A patient who was diagnosed with a systemic disease that was not detected in the pre-clinical screening;
(2) If a patient was confirmed to be pregnant during the treatment period;
(3) If the patient withdrew his/her consent to participate in the clinical trial;
(4) If the patient was lost to follow-up;
(5) Other reasons for which a patient was deemed not appropriate to continue with study participation according to the judgment of the responsible physician.

### 2.6. Concomitant Treatment

During the treatment period or follow-up period of this study, for patients with severe post-accident syndrome, medications and treatment prescribed at other medical institutions were allowed for the treatment and alleviation of the symptoms. In this case, the content and frequency of the concomitant treatment were recorded in detail in the case report form (CRF) and used for analysis.

### 2.7. Outcome Measures

All surveys were conducted at the time of study enrollment, at the mid time point of the treatment visit period, immediately after the end of the treatment visit period, and at follow-up, and these were conducted by KMDs who had been trained in advance regarding the method of analysis in this trial to obtain consistent measurement results. 

#### 2.7.1. Primary Outcome: Numeric Rating Scale of Overall Post-Accident Syndromes

The severity of post-accident syndrome was assessed using the numeric rating scale (NRS), which is a scale in which the patient subjectively indicates the severity of their symptoms; in clinical practice, it has been used for representing the severity of a patient’s somatic pain on a numerical scale [17,18,19,20]. In this study, NRS was used for the numeric representation of not only somatic pain but also the subjective severity of symptoms other than pain. The participant was asked to report the severity of symptoms by selecting from whole numbers from 0 to 10, where 0 indicates ‘no pain or discomfort’ and 10 indicates ‘the most severe pain and discomfort imaginable’. 

#### 2.7.2. Secondary Outcome

1. NRS of musculoskeletal complaints of post-accident syndromes

NRS was used to represent the subjective severity of musculoskeletal symptoms experienced by the patient following the road traffic accident. Participants were asked to rate the severity of pain in each area, such as the neck, lower back, shoulder, and knee, which are the representative musculoskeletal complaints. For pain in other areas of the musculoskeletal system, the areas of the complaint and NRS score were assessed. In this way, the overall severity of musculoskeletal symptoms encompassing pain in the above-described areas was assessed.

2. NRS of neurological complaints of post-accident syndromes

NRS was used to represent the subjective severity of neurological symptoms experienced by the patient following the road traffic accident. The severity of headache and dizziness was specifically assessed, in addition to the overall severity of neurological complaints.

3. NRS of psychiatric complaints of post-accident syndromes

NRS was used to represent the subjective severity of the psychiatric symptoms experienced by the patient following the road traffic accident. The severity of anxiety, depression, anger, and insomnia was specifically assessed, in addition to the overall severity of psychiatric complaints.

4. NRS of digestive systemic complaints of post-accident syndromes

NRS was used for the objective representation of the subjective severity of systemic digestive symptoms experienced by the patient following the road traffic accident. The severity of indigestion and loss of appetite was specifically assessed, and furthermore, the overall severity of the systemic digestive complaints was assessed.

5. NRS of general symptoms of post-accident syndromes

NRS was used to represent the subjective severity of the general symptoms experienced by the patient following the road traffic accident. The severity of fatigue and general weakness was specifically assessed, and furthermore, the overall severity of the general symptoms was assessed.

6. Impact of Event Scale-Revised-Korean

The Impact of Event Scale-Revised-Korean (IES-R-K) is a self-report measure used for the assessment of the subjective stress caused by exposure to a traumatic event and consists of a total of 22 items. Weiss and Marmar revised the original Impact of Event Scale (IES) [21], a 15-item self-report scale developed by Horowitz et al. [22]. The items of the IES-R were designed to measure intrusion, avoidance, and hyperarousal among the most commonly reported patterns of post-trauma psychological responses [23]. Based on the experience of symptoms over the last week, the severity of symptoms was rated on a 5-point scale from 0 (not at all) to 4 (extremely), with a total score ranging from 0 to 88. The scale has been translated into many languages for trauma-related clinical studies worldwide [24,25,26,27]. In particular, it is actively used for investigating and analyzing psychological patterns after motor vehicle accidents [23,28,29,30,31]. 

In this study, the IES-R-K was used as a tool to assess the subjective stress experienced by the participants following road traffic accidents. In this study, the Korean version of IES-R-K, whose reliability and validity were previously verified, was used [32].

7. Five-level EuroQol 5-dimension

The 5-level EuroQol 5-dimension (EQ-5D-5L) is a tool developed by the EuroQol Group [33], and it is a widely used sale in the healthcare sector due to its verified objectivity and validity in the assessment of health-related quality of life [33,34,35,36,37,38]. The EQ-5D-5L consists of five items of multiple-choice questions assessing the current health condition of the participant (mobility, self-care, usual activities, pain/discomfort, and anxiety/depression) and five response levels (1, no problems; 2, slight problems; 3, moderate problems; 4, severe problems; 5, extreme problems) are used for assessment. In this study, the Korean version of the EQ-5D-5L, whose validity was verified in a previous study, was used [39].

8. Short-Form-12 Health Survey version2

The Short-Form-12 Health Survey version2 (SF-12 v2) is a questionnaire designed for the assessment of health-related quality of life (HRQoL). It is an abridged version of the original 36-item health-related quality-of-life survey developed by Ware et al. [40,41,42]. It is a standardized metric that has been used for a long time for the assessment of the general health level of survey participants worldwide [43,44,45,46]. SF-12 is largely divided into two domains: physical health components and mental health components. Physical health components include the following four domains: physical functioning, role limitations due to physical problems, bodily pain, and general health. Mental health components include four domains of vitality, social functioning, role-emotional and mental health [41,42]. It consists of 12 items across the above-described 8 domains, and a high score indicates a better health-related quality of life. In this study, the Korean version of the SF-12 was used, whose reliability and validity were verified in a previous study [47].

9. Work Productivity and Activity Impairment

Productivity loss was measured using the Work Productivity and Activity Impairment (WPAI): Specific Health Problem (WPAI-SHP) questionnaire, which measures absenteeism, presenteeism, and overall work impairment (combined index of absenteeism and presenteeism) due to specific health problems (i.e., road traffic accident) in the past week for employed respondents. Activity impairment (impairment in regular activities) was measured for both employed and unemployed patients [48]. For the analysis of the change in WPAI of unpaid workers, we considered that overall work impairment for paid workers and activity impairment for unpaid workers represented productivity loss.

10. Patient Global Impression of Change

The Patient global impression of change (PGIC) is a scale for the assessment of improvements in post-accident syndromes. Participants subjectively rated the improvement on a 7-point Likert scale (1, very much improved; 2, much improved; 3, minimally improved; 4, no change; 5, minimally worse; 6, much worse; or 7, very much worse).

11. Drug consumption

The type and dose of medications that participants took during the study period were surveyed during their visits. In terms of the type of medications, all drugs that had been prescribed owing to the current disease or those medications that the patients took because of the symptoms related to this study were checked.

12. Adverse events

For an assessment of safety, liver function tests (T-protein, albumin, T-bilirubin, alkaline phosphatase [ALP], alanine transaminase [ALT], aspartate transaminase [AST], gamma-glutamyl transpeptidase [r-GTP]), and renal function tests (blood urea nitrogen [BUN], Creatinine) were performed before and after treatment for both the HM and Control groups. The test results were used to perform between-group comparisons for the incidence of adverse events (AEs). The blood samples obtained from intravenous blood collection were immediately discarded after the analysis of the results, and the process of blood disposal complied with the standard waste disposal guidelines of the diagnostic test team.

AEs are any unfavorable and unintended signs or symptoms or diseases that appear after the treatment, including events that have no causal relationship with the treatment. In this study, information on AEs was collected through symptoms self-reported by patients and the observations of researchers. Furthermore, incidences of AEs suspected of being related to the treatment, abnormal laboratory results, and serious AEs were analyzed.

For the assessment of causality between the treatment and the AEs, a scale with six categories by the World Health Organization-Uppsala Monitoring Center causality assessment system was used (1 = definitely related, 2 = probably related, 3 = possibly related, 4 = probably not related, 5 = definitely not related and 6 = unknown). The severity of all the AEs was classified into 3 categories according to Spilker’s criteria, as follows: Mild (1), symptoms requiring no additional treatment with no functional disruption to the participant’s normal activities of daily living (ADLs); Moderate (2), symptoms causing a significant functional disruption to the participant’s normal ADLs, which may require treatments and disappear over time when additional treatment was applied; Severe (3), symptoms requiring immediate advanced treatment due to severity of the symptoms, resulting in sequelae.

### 2.8. Sample Size Calculation

The null hypothesis of this study was that there would be no difference between the experimental group (HM group) and the control group in the outcomes of post-accident syndrome when IKM treatment and herbal medicine treatment were combined or only IKM treatment was administered for patients with road traffic injuries after the acute phase had elapsed. This study is a pilot study, and the results reported in a previous study [49] were considered, which provided recommendations for the sample size of a pilot study of a minimum of 15 participants per group with a medium target effect size. Assuming a dropout rate of 25%, the target sample size was a total of 40 participants.

### 2.9. Recruitment

In the process of the recruitment of the study participants, necessary information for the study was explained to the outpatients visiting Bucheon Jaseng Hospital of Korean Medicine with complaints of post-accident syndrome, and for those who expressed their wish to participate in the study, the researcher obtained written consent from the patient for enrollment in the study. 

### 2.10. Randomization and Allocation Concealment

Randomization was conducted by a statistics expert using a random number table generated by R studio 1.1.463 (© 2009–2018 RStudio, Inc. Boston, MA, USA) for randomized allocation into the HM (*n* = 20) and Control groups (*n* = 20). Block randomization was used for random sequence, and the size of one block was randomly set to 2 or 4. The results of randomization generated were sealed in opaque envelopes and stored in a double-locked cabinet by a third party not related to the study. The sealed randomization envelope was opened only for those who were eligible according to the inclusion/exclusion criteria and who were sufficiently briefed about the clinical trial by a researcher and gave their voluntary consent by signing a consent form so as to assign the randomization number and enrollment number. The researcher recorded the randomization number assigned to each participant on the electronic chart.

### 2.11. Blinding

As the blinding of the participants and physicians to the group assignment was not possible due to the nature of the interventions, only a single blinding was performed for the researcher that conducted the assessment. This outcome researcher, blinded to the assignment of the treatment groups and who did not participate in the treatment, assessed the participants in a separate space.

### 2.12. Data Collection and Management

In this study, Microsoft Excel CRF was used. The range check for the data values was conducted by issuing a query. Data entered into Excel CRF was locked after completing the data cleaning process, and the access was blocked for all researchers except the person in charge of the data.

### 2.13. Statistical Analysis

In this study, Intention-To-Treat (ITT) analysis was performed. The sociodemographic characteristics of the patients participating in the study were evaluated for each group. Continuous variables are expressed as mean (standard deviation), and between-group comparisons of continuous and categorical variables were performed using an independent *t*-test and chi-square test or Fisher’s exact test, respectively.

Analysis of the repeatedly measured changes from baseline for the primary outcome measure (NRS) and secondary outcome measures (NRS, IES-R-K, EQ-5D-5L, and SF-12) was performed using a linear mixed model with baseline value and days from the onset as the covariate, time as the categorical value, and the group as the fixed factor. For sensitivity analysis, analysis of covariance (ANCOVA) was performed by processing the missing values with multiple imputations and last observations carried forward. The values for each time point were presented as least square estimates. In addition, areas under the curve (AUC) between the baseline and end of follow-up (Week 17) were calculated by the trapezoidal rule and compared through ANCOVA. Moreover, a survival analysis was performed with the event as the “recovery of patients with ≥50% reduction in NRS of overall post-accident syndromes.” For all analyses, the significance level was set to 0.05, and all statistical analyses were performed using R version 4.1.1 (© R Foundation for Statistical Computing, Vienna, Austria).

### 2.14. Ethics Approval

The study protocol was approved by the Institutional Review Board (IRB) of the Jaseng Hospital of Korean Medicine (IRB No.: JASENG 2021-05-021) prior to the recruitment of the study participants and was registered at Clinicaltrials.gov (NCT05494008). Information concerning the study institution and the researchers (investigators for the clinical trial) can be found at the trial registration site. Before the start of the study, the researcher in charge of the clinical trial submitted the protocol, CRF, ICF, etc., of this study to the IRB of the study site for approval. Changes in the protocol, CRF, and ICF were made upon obtaining approval from the IRB. To protect the patients participating in the clinical study, all of the researchers participating in this study were educated about the tenets of the Helsinki Declaration, the Korean Good Clinical Practice Guidelines, and the study protocol.

### 2.15. Informed Consent

Before the start of the clinical study, the researcher fully explained the content and treatment of the clinical trial to the participant, and after obtaining the ICF, which was voluntarily signed by them, a copy of the ICF was provided to the participant. As the collection of personal information was necessary for the reimbursement of transportation expenses, the necessity for this was explained to the study participant, and consent related to the collection of personal information was obtained.

### 2.16. Confidentiality

All personal information and data of the patients participating in this study were strictly managed under the supervision of the IRB, and the confidentiality and protection of the participants’ personal information were ensured. All of the data collected from the participants who provided their consent to participate in this study were handled anonymously, and when provided to other institutions for research, the data were provided in the form of a de-identified code excluding personal information.

### 2.17. Ancillary and Post-Trial Care

In the event that the study participants had any questions or experienced medical problems or illnesses related to the disease being investigated during the study period, emergency contact information was provided to them such that they could receive necessary treatments or measures by contacting the principal investigator or one of the investigators. The herbal medicine prescribed in this study is the same as that prescribed in real clinical practice, and the safety of the prescribed herbal medicine has been secured through years of clinical experience; these were prepared at Jaseng Herbal Dispensary, certified by the Ministry of Health and Welfare. If adverse reactions considered to be related to the herbal medicine taken in the study occurred, immediate medical actions (such as the discontinuation of the medicine and additional prescription of supplementary herbal medicine/adjunct agent) were taken. In the case of direct injury related to this study, the study participants underwent tests and received treatments that were deemed appropriate according to the standard treatment guidelines and based on the decisions of the researcher in charge. Additionally, a plan to compensate participants was prepared according to the agreement of indemnification and insurance policy conditions. 

## 3. Results

### 3.1. Participants Flow

**Figure 1:**
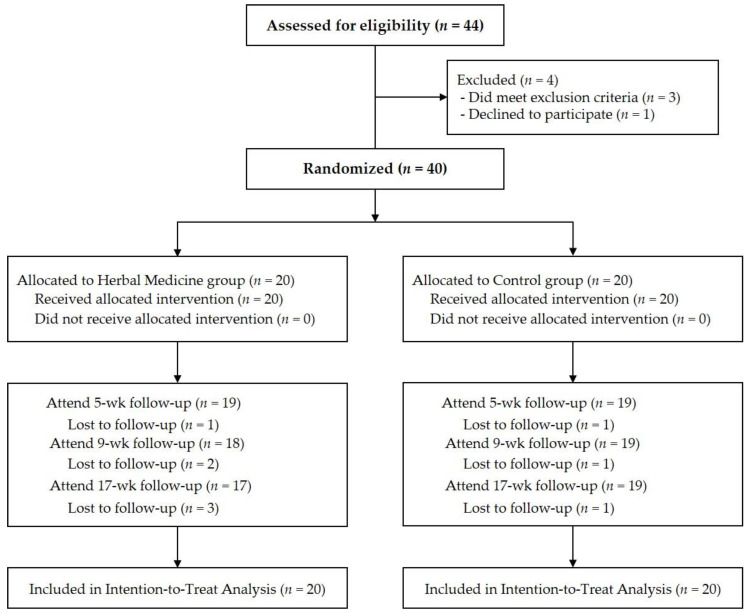
Flow chart of patients of the total of 44 patients screened, 40 who met the inclusion criteria and agreed to participate in the study were enrolled in the study. Intention-to-treat analysis was performed on 20 herbal medicine group and 20 control.

A total of 44 patients were screened from July 2021 to May 2022. Among them, three patients were eliminated because they met the exclusion criteria, one patient withdrew consent just before randomization, and finally, 40 patients were enrolled in the study. Forty patients were randomized to the HM group (*n* = 20) and Control group (*n* = 20). After randomization, three patients from the HM group were lost-to-follow up, one during the treatment period who did not visit the hospital without any notice on the scheduled visit and did not respond to multiple phone calls and text messages from the researcher and two during the follow-up. One patient from the control group withdrew from study participation during the treatment period, whose consent to participate in the study was withdrawn for unknown reasons. However, ITT analysis was performed with all 20 patients in the HM group and all 20 in the control group (Figure 1).

### 3.2. Baseline Characteristics

Regarding baseline characteristics of the HM group (*n* = 20) and control group (*n* = 20), there were no significant differences between the two groups for each of the characteristics, except for “the days from onset” (Table 1). The mean age of participants was 37.4 ± 8.8 years in the HM group and 42.0 ± 10.5 years in the control group, and the male-to-female ratio was similar between the two groups. As for the type of road traffic accident, the proportion of accidents that occurred while the participant was outside the car was high in both groups. The time elapsed from the date of the accident was 90.9 ± 34.5 days in the HM group and 69.0 ± 18.4 days in the control group. Thus, although the participants were allocated to groups by randomization, “days from onset” was significantly longer in the HM group than that in the Control group. The number of participants diagnosed with cervical and lumbar herniated intervertebral disc (HIVD) was seven in the HM group and ten in the Control group. The number of participants who were diagnosed with HIVD before the traffic accident was one in the HM group and two in the Control group. There were nine patients in the HM group and five patients in the control group whose HIVD status could not be determined because in-depth examinations were not carried out before or after the traffic accident.

The overall score of post-accident syndrome (NRS) was 6.3 ± 0.9 in the HM group and 6.3 ± 0.7 in the Control group, showing similarity between the two groups. The scores for the Impact Event Scale (IES-R-K), a scale for the assessment of subjective distress specific to the road accident, were 27.3 ± 18.6 in the HM group and 20.0 ± 16.8 in the Control group, indicating a slightly higher score in the HM group, but the difference between the two groups was not significant (Table 1). Table 2 presents detailed information on the types and severity of post-accident syndromes complained of by the patients (Table 2). 

### 3.3. Treatment

Both the HM and the control groups received the following IKM treatments: acupuncture, pharmacoacupuncture, moxibustion, cupping, Chuna manual therapy, Doin therapy, and Interferential current therapy (ICT). All patients in the HM group underwent acupuncture and cupping, and the average number of treatments during the study period was 6.60 ± 1.88 and 6.40 ± 2.23, respectively. Next, pharmacoacupuncture was performed on 19 patients (95.0%), and the average number of treatments was 4.05 ± 1.54. ICT and Doin therapy were administered to a total of 12 patients (60.0%), and the average number of treatments was 4.05 ± 3.75 and 3.70 ± 3.44, respectively. Moxibustion was administered to six patients (30.0%), and Chuna manual therapy was administered to three patients (15.0%); the average number of treatments was 2.25 ± 3.55 and 0.45 ± 1.23, respectively. All patients in the control group underwent acupuncture, cupping, and pharmacoacupuncture and the average number of treatments was 5.40 ± 1.64, 5.35 ± 1.76, and 4.25 ± 1.02, respectively. This was followed by ICT, which was performed on 13 patients (65.0%), and the average number of treatments was 3.65 ± 2.98. Doin therapy and moxibustion were administered to eight patients (40.0%), and the average number of treatments administered was 2.15 ± 2.92 and 1.85 ± 2.58, respectively. Chuna manual therapy was administered to 10 patients (50.0%), and the average number of treatments was 1.45 ± 1.93 (Supplementary Table S2).

A total of 29 types of herbal medicine were prescribed to the patients in the HM group. Yukgong-dan (六拱丹) was the most prescribed herbal medicine, with 12 prescriptions for patients (60.0%), and the average number of prescription days was 23.33 ± 6.89. Gwanjeol-go (關節膏) was the next most prescribed herbal medicine, which was prescribed to nine patients (45.0%), and the average number of prescription days was 23.33 ± 7.00. This was followed by Chungpa-Jeon-H, Gamiseogyeong-tang (加味舒经湯), Yukgongbaro-hwan, and Chungpajeonsinbang 2 (Supplementary Table S3).

### 3.4. Primary and Secondary Outcomes

**Figure 2:**
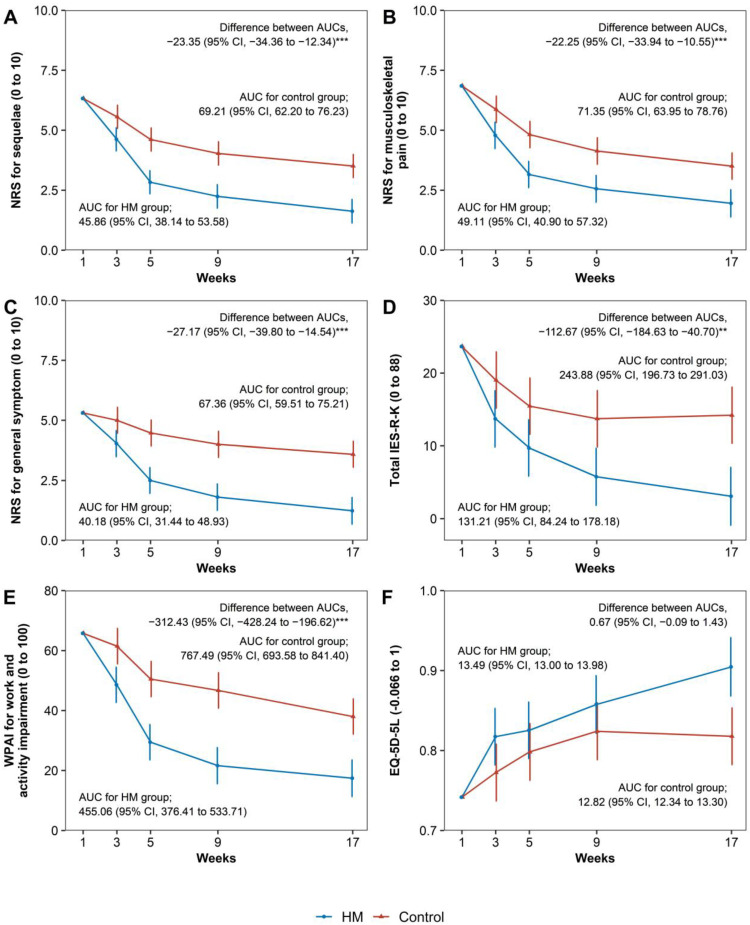
Changes in outcomes over time and areas under the curves. (**A**) NRS score for overall post-accident syndromes; (**B**) NRS score for musculoskeletal complaints of post-accident syndromes; (**C**) NRS score for general symptoms of post-accident syndrome; (**D**) IES-R-K-total score; (**E**) WPAI for work and activity impairment; (**F**) EQ-5D-5L score. The dots show the mean scores, and the error bars show the 95% confidence intervals. All values are presented with least square estimates and its confidence interval. NRS, numeric rating scale for symptom used by patients to report their symptom level as a number from 0 (no symptom) to 10 (most severe symptom imaginable); IES-R-K, impact of event scale-revised-Korean with scores calculated by the severity of symptoms on a 5-point scale from 0 (not at all) to 4 (extremely) based on the past week, with an overall total score of 0–88; EQ-5D-5L, EuroQoL 5-dimension 5-level instrument with scores calculated by converting the patients’ responses to a scale ranging from −0.066 (lowest quality of life) to 1 (highest quality of life). ** *p* < 0.01, *** *p* < 0.001.

The outcome values of the HM group and control group at each time point and the difference in outcome between the two groups were analyzed using a linear mixed model. At the primary endpoint of week 5, the outcomes of the HM group showed statistically significant improvements over the Control group in the following outcome measures: NRS of overall post-accident syndromes, NRS of musculoskeletal complaints of post-accident syndromes, NRS of neurological complaints of post-accident syndromes, NRS of psychiatric complaints of post-accident syndromes, NRS of general symptoms of post-accident syndromes, IES-R-K-avoidance, IES-R-K-total, and WPAI (work and activity). At Week 9 in the follow-up period, there was a statistically significant difference between the two groups, with the HM group continuing to show superior improvement to that of the Control group. At Week 17 in the follow-up, the outcomes of the HM group demonstrated statistically significant improvements over the Control group in the following outcome measures: NRS of overall post-accident syndromes, NRS of musculoskeletal complaints of post-accident syndromes, NRS of neurological complaints of post-accident syndromes, NRS of psychiatric complaints of post-accident syndromes, NRS of general symptoms of post-accident syndromes, IES-R-K-hyperarousal, IES-R-K-avoidance, IES-R-K-intrusion, IES-R-K-sleep problem and numbness, IES-R-K-total, WPAI (work and activity), EQ-5D-5L, and PCS (Table 3, Figure 2). Similar results were obtained in the sensitivity analysis using ANCOVA. (Supplementary Tables S4 and S5).

In the results of the analysis for 17-week outcomes based on the calculation of AUC, statistically significant effects were shown in the HM group compared to those of the control group in the following outcome measures: NRS of overall post-accident syndromes, NRS of musculoskeletal complaints of post-accident syndromes, NRS of neurological complaints of post-accident syndromes, NRS of psychiatric complaints of post-accident syndromes, NRS of general symptoms of post-accident syndromes, IES-R-K-hyperarousal, IES-R-K-avoidance, IES-R-K-intrusion, IES-R-K-sleep problem and numbness, IES-R-K-total, and WPAI (Table 4). 

### 3.5. Survival Analysis

**Figure 3:**
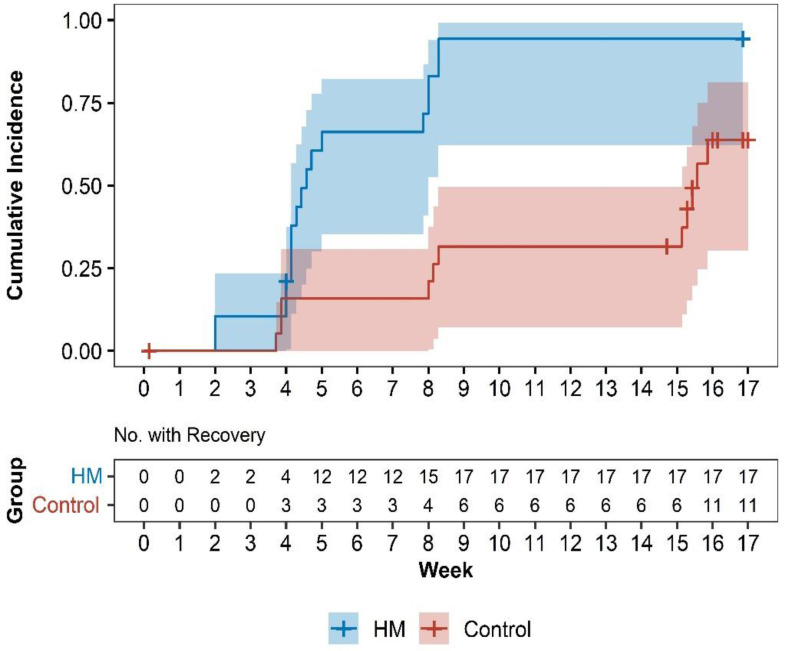
Cumulative incidence curves of recovery by group. Survival analysis was performed with the event of “recovery of patients with ≥50% reduction in NRS of overall post-accident syndromes”. HM, herbal medicine; NRS, numeric rating scale; HR, hazard ratio; CI, confidence interval.

In a survival analysis based on the recovery criteria of “patients with a reduction in NRS of overall post-accident syndromes ≥ 50%,” the time to recovery was shorter in the HM group than that in the control group during the 17-week study period (*p* < 0.001 by the log-rank test). The median time to recovery measured within 17 weeks was 32 days after randomization in the HM group (95% CI: 29–56) and 109 days after randomization in the control group (95% CI: 106-NA). The hazard ratio for the number of patients with a decrease in NRS of overall post-accident syndromes of ≥50% was 3.40 (95% CI: 1.37–8.45) at 17 weeks, favorable to the HM group (Figure 3).

### 3.6. Drug Consumption

During the study period, there were two patients (10.0%) in the HM group and seven patients (35.0%) in the control group who took medications related to post-accident syndrome during the study period, and the average number of prescription days was 1.0 ± 0.0 and 7.6 ± 6.2, respectively. One of two patients in the HM group received an analgesic injection at a Western hospital for relief of lower back pain during the treatment period, and the other patient in the HM group took NSAIDs (Ibuprofen) once due to a headache during the follow-up period. Seven patients in the control group took NSAIDs (Aceclofena, Naproxen, Ppopionic acid derivatives, etc.), skeletal muscle relaxants (Eperisone), and acetaminophen, etc., as a means of pain relief for post-accident lower back pain, back pain, and headaches (Supplementary Table S7). 

### 3.7. Adverse Events

Adverse events (AEs) occurred in twelve patients in the HM group and nine patients in the control group. AEs determined to be related to the treatment received occurred in five patients in the HM group but none in the control group. Most of the AEs were digestive symptoms with diarrhea in three patients, a feeling of fullness in the abdomen in one patient, and heartburn, chest tightness, and nausea in one patient. Among those who complained of diarrhea, two out of three patients showed an improvement without any treatment, and one patient was prescribed supplementary herbal medicine and recovered within two days after taking the newly prescribed medicine. One patient who complained of a feeling of fullness in the abdomen declined an additional prescription because the severity of the symptom was mild and recovered naturally within 4 days. In the case of a patient who intermittently complained of transient heartburn, chest tightness, and nausea while taking herbal medicine, the developed symptoms were mild, and they disappeared within 1 to 2 days. Supplementary herbal medicine was prescribed for chest tightness and nausea, and the patient took the medicine accordingly. 

In the blood tests performed before and after the treatment period, a total of 16 participants showed 21 abnormal lab values after the completion of the treatment (10 participants in the HM group and six participants in the Control group). Among these participants, 11 patients had abnormal findings even before the treatment, with seven participants in the HM group (two each had abnormal T-bilirubin and ALT values and one each had abnormal BUN, ALP, r-GTP values) and four participants in the Control group (two each had abnormal T-bilirubin and BUN values and two each had abnormal ALT values). There were seven participants in total who showed test results of normal range prior to the treatment but reported abnormal findings after the treatment, consisting of four participants in the HM group (one each had abnormal creatinine, ALP, and r-GTP values and two each had abnormal AST and ALT values) and three participants in the Control group (one each had abnormal T-bilirubin, creatinine, and r-GTP values). On the contrary, five participants (one case each of abnormal T-bilirubin and creatinine values and two each of abnormal BUN and ALT values) and seven participants (three each had abnormal BUN values and two each had abnormal T-bilirubin values, one each had abnormal creatinine and ALP values) showed abnormal findings before treatment but normal findings after treatment. 

## 4. Discussion

The present study investigated the safety and efficacy of Korean herbal medicine in patients with whiplash injuries post-accident. We made the following observations: (i) IKM treatment with herbal medicine treatment showed significant improvement in outcome measures for post-accident syndrome (NRS), Impact Event Scale, productivity, and quality of life for patients after the acute phase compared to that for IKM treatment alone without herbal medicine after 4 weeks of treatment; and (ii) this effect lasted even at follow-up time points of Week 9 and Week 17.

In modern society, road traffic accidents are one of the leading causes of death and other disabilities [50]. However, there are many cases in which, even with complaints of severe pain and functional impairment, the cause of the pain cannot be identified through diagnostic tests [15]. Among the road traffic casualties, there are cases of patients with bruises and sprains whose diagnostic radiographs show no surgical abnormalities in particular, but complaints of pain and discomfort persist even several months after the accident. These patients report not only somatic pain of the affected site but general symptoms other than pain, such as anxiety or depression, insomnia, dizziness, or gastrointestinal symptoms [5,6]. Such a post-accident syndrome persists for more than 3 months in 66.4% of patients with road traffic injuries [4], and this causes a significant compromise in the quality of life compared to the pre-trauma level and increased social burden of medical costs [51]. Therefore, it is imperative that post-accident syndrome of patients with road traffic injuries is properly managed.

Although the automobile insurance system in South Korea is privatized, automobile insurance is mandatory for all drivers purchasing a car and is managed by the Health Insurance Review and Assessment Service (HIRA) on a national level. In the event of a road traffic accident, the inflictor’s insurance company pays the victim’s medical expenses, and the insurance payment of a motor vehicle accident is processed according to the degree of negligence in the accident, the traffic accident guidelines of the Ministry of Land, Infrastructure and Transport, and the policies of the Auto Insurance Medical Fee Review Council under HIRA. At present, under the automobile insurance system of South Korea, there are some restrictions regarding treatment options, the number of treatments, and diagnostic tests [15]. In South Korea, in the dual healthcare delivery system of Western and Korean medicine, the option of Korean medicine treatment is guaranteed on a governmental level within the automobile insurance system. Therefore, both Western and Korean medicine treatment options for post-accident syndrome are available to the public. According to the Automobile Insurance Medical Expenses Statistics in 2022 [52] published by HIRA, the number of patients treated at Korean medicine hospitals and clinics for post-accident syndrome for 5 years from 2017 showed an annual average increase of 8.65% and 1.30%, respectively. The proportion of medical expenses also increased considerably in the Korean medicine sector, with an average increase of 18.70% annually for 5 years from 2017. Through the statistics, it can be seen that there is a growing importance regarding the role of Korean medicine institutions in the treatment and management of patients with road traffic injuries, and the preference and level of trust of patients for Korean medicine have also increased.

Examining the baseline characteristics of the patients who participated in this study, the mean age was in the late 30 s for the HM group and the early 40 s for the control group, and the male:female ratio was 35:65 for the HM group and 45:55 for the Control group. Most of the participants were recruited approximately 2 to 3 months from the date of the accident, and the NRS for the overall post-accident syndrome was found to have an average value of 6.3 in both groups, indicating that although the acute phase had elapsed, a post-accident syndrome of moderate severity was reported by these patients. In this study, at the primary endpoint of Week 5, the outcomes of the HM group showed statistically significant improvements over the Control group in the following outcome measures: NRS of overall post-accident syndromes, NRS of musculoskeletal, neurological, psychiatric complaints of post-accident syndromes, NRS of general symptoms of post-accident syndromes, IES-R-K-avoidance, IES-R-K-total, and WPAI (work and activity). The results indicate that herbal medicine treatment played a significant role in the improvement in the overall post-accident syndrome, such as mental and psychological symptoms, including somatic pain caused by the road accident, and in the recovery from the loss of productivity due to the accident. In a survival analysis based on the recovery criteria of “patients with a reduction in NRS of overall post-accident syndromes of ≥50%,” the time to recovery was shorter in the HM group than that in the control group during the 17-week study period. That is, when IKM treatment combined with herbal medicine treatment was administered, patients showed faster improvement in the post-accident syndrome compared to those that received the intervention without herbal medicine treatment, and the treatment effect continued up to 17 weeks with a significant difference from the Control group. In addition, in a total of five cases of AEs in the HM group, the symptoms were mild and improved within 1 to 7 days with a supplemental prescription or without additional treatment. Therefore, it can be considered that there is a low risk of serious or severe adverse events due to the administration of herbal medicine, and thus, combined therapy with herbal medicine can be judged as a safe treatment strategy.

The most commonly prescribed herbal medicine in this study was Yukgong-dan (六拱丹), followed by Gwanjeol-go (關節膏), Chungpa-Jeon-H, and Gamiseogyeong-tang (加味舒经湯) in that order. The main ingredients of Yukgong-dan (六拱丹) are Rehmanniae Radix, Angelica gigas Nakai, Aquilariae Lignum, etc. Rehmanniae Radix (地黃) has been reported to have the effect of weakening pain sensitization and improving bone quality through the regulation of signal transduction pathways [53], and thus, it is expected to have the effect of relieving somatic pain from the post-accident syndrome. In the case of Angelica gigas Nakai (當歸), decursinol, a coumarins derivative extracted from the root, was reported to be effective in pain relief by antinociception [54]. Aquilariae Lignum (沈香) was found to have analgesic and anti-inflammatory properties for pain and inflammatory conditions in an in vivo experiment [55]. The main ingredients of Gwanjeol-go (關節膏) include Panax ginseng C.A. Meyer, Achyranthis Radix, Poria cocos Wolf, and Rehmanniae Radix. Panax ginseng C.A. Meyer (人蔘) is a medicinal herb with extensive pharmacological activities and efficacy, and it has a long history of use for improvement in neurological diseases, such as insomnia, depression, anxiety and nervous breakdown, in traditional Chinese and Korean medicine [56]. Ginsenoside, a main ingredient of Panax ginseng C.A. Meyer, has been reported to have an antidepressant effect by alleviating the dysfunction of monoamine transmitters and modulating neuroendocrine dysfunction [56,57]. Achyranthis Radix (牛膝) has been reported to be effective at preventing bone loss through potent inhibitory activity on bone resorption through parathyroid hormone [58] and to accelerate peripheral nerve regeneration [59]. Two polysaccharides (PCWPW (37,154 Da) and PCWPS (186,209 Da)) extracted from Poria cocos Wolf (茯笭) were shown to have an obvious antidepressant effect in an animal model [60].

Constituents of Gamiseogyeong-tang (加味舒經湯) include Curcumae Longae Rhizoma, Angelica gigas Nakai, and Paeoniae Radix rubra. Anti-inflammatory, analgesic, and neuroprotective effects of Curcumae Longae Rhizoma (薑黃) were demonstrated in inflammatory disorders and pain [61]. Paeoniae Radix rubra (赤芍藥) was reported with its neuroprotective, analgesic, sedative, and antidepressant effects [62,63]. Chungpa-Jeon H, Chungpa-Jeon, and Chungshinbaro-hwan were prescribed for improvement in musculoskeletal complaints among post-accident syndrome, and all of the herbal medicine has GCSB-5 (a purified extract from a mixture of six oriental herbs) as the main ingredient. The ingredients of GCSB-5 include Acanthopanacis Cortex (五加皮), Achyranthes bidentata Blume (牛膝), Saposhnikovia divaricata Schiskin (防風), Eucommia ulmoides Oliver (杜沖), Cibotium barometz J. Smith (狗脊) and Glycine max Merrill (大豆); it has been reported to reduce acute and chronic inflammation, protect articular cartilage and to have a potent analgesic effect [64,65]. Gamidokhwalgisaeng-tang (加味獨活寄生湯) is prescribed with its ingredients of Saposhnikovia divaricata Schiskin, Acanthopanacis Cortex, and Achyranthis Radix, and it has been shown to be effective in the treatment of osteoarthritis by inhibiting chondrocyte apoptosis [66]. Eleutherosides, the active constituent of Acanthopanacis Cortex (五加皮), is known to reduce the accumulation of lactic acid in muscles and protect the muscle tissue [67]. Gamisoyo-san (加味逍遙散), which was prescribed for improvement in psychiatric symptoms, such as anxiety, depression, and insomnia, after the road traffic accident, was shown to be effective in rapid improvement of anxiety and enhancing sleep quality in a clinical study for patients with mild to moderate depression with anxiety symptoms [68]. 

In conclusion, patients who complain of physical pain remaining after the acute phase are believed to have alleviated by blood circulation, anti-inflammatory, and pain relieving mechanisms of Rehmanniae Radix, Curcumae Longae Rhizoma, Angelica gigas Nakai, Aquilariae Lignum, and GCSB-5, etc. In addition, various psychological symptoms such as anxiety/depression that could last after the psychological shock of an accident would be alleviated through sedation and the antidepressant mechanism of Panax ginseng C.A. Meyer, Poria cocos Wolf, and Gamisoyo-san. 

Five subjects in the HM group complained AEs that were judged to be related to treatment, such as diarrhea, heartburn, and nausea. Since they took a herbal medicine, which is a combination of medicinal herbs, it is difficult to clearly identify which action is due to which medicinal herb. However, for the pharmacological effects of the chemical components of a few single herbs, it seems possible to infer an association with AEs. Rehmanniae Radix (地黃), which belongs to the composition of many of the herbal medicines used in this study, contains a large amount of stachyose and raffinose, which are non-digestible sugars that do not have digestive enzymes in the human body. The ingestion of these non-digestible sugars is known to cause diarrhea, bloating, and abdominal pain [69,70], so it can be inferred that they are related to the side effects reported in this study. Curcumae Longae Rhizoma (薑黃) is also a medicinal herb with a long-established safety record, but it is thought to be related as it has been reported that diarrhea or nausea occurs as common side effect of high-dose intake [71].

All of the symptoms of the subjects who complained of AEs were mild enough for recovery with short-term supplementary herbal medicine or disappeared without any supplementary measures. On the other hand, in terms of the effect of improving the post-accident syndromes following the intake of herbal medicine, it is judged that the benefit obtained by taking the herbal medicine was greater than the AEs.

The limitations of this study are as follows. Since this study was designed and conducted as a pilot study, the sample size was small. A small sample size induces an imbalance in the treatment provided between two groups despite randomization. The percentage of patients who received Chuna manual therapy was much smaller in the HM group than in the control group. However, this may not be a limitation of this study, but rather a strength, because it means that the effect of HM treatment analyzed in this study may have been underestimated. In addition, since it fulfilled the requirements for the minimum sample size to be recruited to obtain valid results, as suggested by a previous study [49], we could draw results with significant implications, thereby justifying the rationale and the necessity of this study. Future studies with an increased number of patients are needed. Second, because the interventions of the HM group and the Control group were different, the blinding of the KMDs and the study participants was not possible. Therefore, the placebo effect cannot be completely excluded from the results of this study. The placebo effect can be controlled to some extent by having the control group take a drink with a taste and color similar to herbal medicine, but this could not proceed due to various constraints such as cost, technology, and time. Nevertheless, to minimize the bias, the researchers evaluating the outcomes were blinded to the group assignments and did not participate in the treatment. Third, in this study, the effect of herbal medicine treatment as a monotherapy could not be identified. However, in clinical practice, IKM treatment and herbal medicine treatment are administered simultaneously; therefore, this study can be regarded as a pragmatic study reflecting real-world clinical practice. It will be necessary to design and conduct a randomized clinical trial to evaluate the effectiveness of herbal medicine alone.

The significant contribution of this study is that this is the first pragmatic clinical study of herbal medicine treatment for post-accident syndrome after the acute phase has elapsed. In this study, we recruited a wide range of patients who complained of symptoms severe enough to cause discomfort in daily life even after more than 8 weeks had elapsed from the date of the accident. Additionally, in this study, the physician was not asked to provide a specific treatment, nor were there any limitations on the types of herbal medicines that were prescribed. Accordingly, the physicians were able to autonomously prescribe herbal medicine and perform treatments based on their clinical judgment and according to the patient’s symptoms, which is very similar to the actual practice in Korean medicine, highlighting the pragmatic nature of this study.

The results demonstrate the effectiveness and safety of the herbal medicine treatment strategies widely used in the clinical practice of Korean medicine for the treatment and management of patients with road traffic injuries. Thus, we believe that our findings will present important evidence to inform future decision-making policies as well as to further the clinical evidence database.

## 5. Conclusions

Compared to IKM treatment alone, IKM treatment in combination with herbal medicine treatment showed a significant decrease in the NRS score of post-accident syndrome persistent after the acute phase. In particular, the musculoskeletal, neurological, and psychiatric complaints and general symptoms of post-accident syndromes were significantly reduced. The degree of psychological shock and loss of productivity of the patients were also improved, resulting in a significant improvement in quality of life.

## 6. Protocol Version

The study protocol version is 1.4 (6 June 2022). Major revisions of the study protocol and other changes made after this paper will be updated at the trial registration site.

## Figures and Tables

**Table 1:** Baseline characteristics of the participants.

	HM Group	Control Group	*p*-Value
	(N = 20)	(N = 20)	
Sex			
Female	7 (35.0)	9 (45.0)	0.747
Male	13 (65.0)	11 (55.0)	
Age (year)	37.4 ± 8.8	42.0 ± 10.5	0.137
Height (cm)	171.5 ± 7.4	169.4 ± 8.0	0.396
Weight (kg)	74.5 ± 14.7	71.5 ± 13.1	0.505
BMI (kg/m^2^)	25.2 ± 4.0	24.8 ± 3.4	0.726
Days from onset	90.9 ± 34.5	69.0 ± 18.4	0.017 **
Type of traffic accident			
Inside the car	4 (20.0)	1 (5.0)	0.341
Outside the car	16 (80.0)	19 (95.0)	
HIVD			
Yes	7 (35.0)	10 (50.0)	0.437
No	4 (20.0)	5 (25.0)	
Unknown	9 (45.0)	5 (25.0)	
Credibility and expectancy of improvement			
With herbal medicine	7.0 ± 1.2	6.7 ± 1.3	0.453
Without herbal medicine	6.0 ± 1.3	5.4 ± 1.0	0.176
NRS score for overall post-accident syndromes	6.3 ± 0.9	6.3 ± 0.7	0.852
IES-R-K			
IES-R-K-total	27.3 ± 18.6	20.0 ± 16.8	0.201
IES-R-K-hyperarousal	7.5 ± 5.7	5.0 ± 4.5	0.142
IES-R-K-avoidance	7.2 ± 5.7	5.6 ± 6.0	0.380
IES-R-K-intrusion	7.1 ± 4.9	5.2 ± 4.9	0.240
IES-R-K-sleep problem and numbness	5.5 ± 3.8	4.2 ± 3.3	0.238
WPAI (Employment status)			
Yes	17 (85.0)	20 (100.0)	0.231
No	3 (15.0)	0 (0.0)	
WPAI (absenteeism)	10.2 ± 19.1	3.1 ± 11.4	0.171
WPAI (presenteeism)	61.8 ± 7.3	63.5 ± 7.5	0.48
WPAI (activity impairment)	64.0 ± 8.8	63.5 ± 7.5	0.848
WPAI (work and activity impairment)	67.1 ± 10.3	64.4 ± 8.9	0.389
EQ-5D-5L	0.72 ± 0.13	0.77 ± 0.10	0.184
SF-12 (PCS)	39.7 ± 7.4	42.2 ± 8.1	0.313

The data are expressed as mean ± standard deviation (SD) or number (%). The between-group comparisons of the continuous and categorical variables were performed using an independent *t*-test and the chi-square test or Fisher’s exact test, respectively. HIVD, herniated intervertebral disc; HM, herbal medicine; NRS, numeric rating scale for symptom used by patients to report their symptom severity as a number from 0 (no symptom) to 10 (most severe symptom imaginable); IES-R-K, impact of event scale-revised-Korean with scores calculated by the severity of symptoms on a 5-point scale from 0 (not at all) to 4 (extremely) based on the past week, with an overall total score of 0–88; WPAI, work productivity and activities impairment; EQ-5D-5L, EuroQoL five-dimension five-level instrument with scores calculated by converting the patients’ responses to a scale ranging from −0.066 (lowest quality of life) to 1 (highest quality of life); SF-12, 12-item short-form health survey with scores calculated by converting the patients’ responses to a scale ranging from 0 (lowest quality of life) to 100 (highest quality of life); PCS, physical component summary; MCS, mental component summary. ** *p* < 0.01.

**Table 2:** Types and severity of complaint symptoms.

	HM Group (*n* = 20)		Control Group (*n* = 20)		*p*-Value
	N (%)	Mean ± SD	N (%)	Mean ± SD	
Musculoskeletal complaints	20 (100.0)	6.8 ± 1.0	20 (100.0)	7.0 ± 1.1	0.563
Neck	18 (90.0)	6.2 ± 1.4	19 (95.0)	5.9 ±1.3	
Lower back	20 (100.0)	6.0 ± 1.4	19 (95.0)	6.4 ± 1.3	
Shoulder	11 (55.0)	4.9 ± 2.3	16 (80.0)	5.2 ± 1.7	
Knee	11 (55.0)	5.4 ± 2.1	9 (45.0)	3.7 ± 1.6	
Neurological complaints	12 (60.0)	4.3 ± 2.1	13 (65.0)	4.3 ± 1.3	0.972
Headache	10 (50.0)	4.8 ± 2.3	11 (55.0)	4.5 ± 1.4	
Dizziness	5 (25.0)	4.0 ± 1.9	9 (45.0)	4.0 ± 0.5	
Psychiatric complaints	17 (85.0)	5.5 ± 2.0	17 (85.0)	5.2 ± 1.6	0.581
Anxiety	14 (70.0)	5.8 ± 1.6	14 (70.0)	4.6 ± 2.2	
Depression	9 (45.0)	5.1 ± 1.5	10 (50.0)	4.7 ± 1.9	
Anger	10 (50.0)	4.8 ± 2.0	10 (50.0)	4.4 ± 2.2	
Insomnia	12 (60.0)	5.5 ± 2.5	14 (70.0)	5.1 ± 1.6	
Digestive systemic complaints	8 (40.0)	4.5 ± 2.1	10 (50.0)	3.2 ± 1.9	0.197
Indigestion	8 (40.0)	4.6 ± 2.4	7 (35.0)	3.3 ± 1.7	
Loss of appetite	3 (15.0)	3.3 ± 1.5	7 (35.0)	3.4 ± 2.4	
General symptoms	18 (90.0)	5.1 ± 1.6	18 (90.0)	5.5 ± 1.8	0.493
Fatigue	18 (90.0)	5.0 ± 1.5	18 (90.0)	5.6 ± 1.8	
General weakness	16 (80.0)	4.6 ± 2.0	13 (65.0)	5.2 ± 1.9	

Data are expressed as mean ± standard deviation (SD) or number (%). Between-group comparisons of numeric rating scales were performed using an independent *t*-test. HM, herbal medicine; NRS, numeric rating scale for symptom used by patients to report their symptom severity as a number from 0 (no symptom) to 10 (most severe symptom imaginable).

**Table 3:** Primary analysis of between–group differences.

		Week 3	Week 5	Week 9	Week 17
NRS score for overall post-accident syndromes	HM group	4.62 (4.14, 5.10)	2.83 (2.35, 3.31)	2.24 (1.76, 2.73)	1.62 (1.13, 2.12)
	Control group	5.56 (5.08, 6.04)	4.61 (4.13, 5.10)	4.04 (3.55, 4.52)	3.51 (3.03, 3.99)
	Difference in decrease	0.94 (0.24, 1.64)	1.78 (1.08, 2.48)	1.79 (1.09, 2.50)	1.89 (1.18, 2.59)
	*p*-value	0.009 **	<0.001 ***	<0.001 ***	<0.001 ***
NRS score for musculoskeletal complaints	HM group	4.79 (4.24, 5.33)	3.15 (2.61, 3.70)	2.56 (2.00, 3.12)	1.95 (1.39, 2.52)
	Control group	5.87 (5.32, 6.42)	4.82 (4.27, 5.37)	4.14 (3.59, 4.69)	3.51 (2.96, 4.06)
	Difference in decrease	1.09 (0.29, 1.89)	1.67 (0.87, 2.46)	1.58 (0.77, 2.38)	1.55 (0.74, 2.36)
	*p*-value	0.008 **	<0.001 ***	<0.001 ***	<0.001 ***
NRS score for neurological complaints	HM group	2.90 (1.94, 3.87)	1.81 (0.85, 2.78)	0.92 (−0.09,1.92)	0.23 (−0.81, 1.27)
	Control group	4.01 (3.12, 4.90)	3.24 (2.35, 4.13)	2.62 (1.74, 3.51)	1.78 (0.89, 2.66)
	Difference in decrease	1.10 (−0.25, 2.46)	1.43 (0.07, 2.78)	1.71 (0.32, 3.09)	1.54 (0.13, 2.96)
	*p*-value	0.107	0.039 *	0.017 *	0.033 *
NRS score for psychiatric complaints	HM group	3.80 (3.11, 4.50)	2.43 (1.73, 3.12)	1.35 (0.63, 2.06)	0.84 (0.10, 1.57)
	Control group	4.74 (4.05, 5.44)	3.99 (3.30, 4.69)	3.24 (2.55, 3.94)	2.74 (2.05, 3.44)
	Difference in decrease	0.94 (−0.07, 1.95)	1.57 (0.56, 2.58)	1.90 (0.87, 2.92)	1.91 (0.87, 2.95)
	*p*-value	0.067	0.003 **	<0.001 ***	<0.001 ***
NRS score for digestive systemic complaints	HM group	2.48 (1.25, 3.71)	0.77 (−0.46, 1.99)	0.05 (−1.17, 1.28)	−0.13 (−1.39, 1.13)
	Control group	1.97 (0.97, 2.97)	1.37 (0.37, 2.37)	1.57 (0.57, 2.57)	1.27 (0.27, 2.27)
	Difference in decrease	−0.51 (−2.22,1.21)	0.61 (−1.11, 2.32)	1.52 (−0.19, 3.24)	1.40 (−0.34, 3.14)
	*p*-value	0.542	0.468	0.079	0.109
NRS score for general symptoms	HM group	4.03 (3.49, 4.57)	2.50 (1.96, 3.04)	1.80 (1.26, 2.35)	1.23 (0.68, 1.79)
	Control group	5.00 (4.46, 5.54)	4.47 (3.93, 5.01)	4.00 (3.46, 4.54)	3.59 (3.05, 4.13)
	Difference in decrease	0.97 (0.19, 1.76)	1.97 (1.19, 2.76)	2.20 (1.41, 2.99)	2.36 (1.56, 3.15)
	*p*-value	0.016 *	<0.001 ***	<0.001 ***	<0.001 ***
IES-R-K-hyperarousal	HM group	3.59 (2.44, 4.74)	2.43 (1.28, 3.59)	1.11 (−0.06, 2.29)	0.65 (−0.54, 1.85)
	Control group	4.92 (3.77, 6.07)	3.76 (2.61, 4.91)	3.66 (2.50, 4.81)	3.18 (2.03, 4.33)
	Difference in decrease	1.33 (−0.35, 3.00)	1.33 (−0.35, 3.00)	2.54 (0.85, 4.23)	2.53 (0.82, 4.24)
	*p*-value	0.119	0.119	0.004 **	0.004 **
IES-R-K-avoidance	HM group	3.50 (1.82, 5.19)	2.93 (1.24, 4.61)	1.20 (−0.51, 2.92)	0.73 (−1.01, 2.47)
	Control group	5.52 (3.83, 7.21)	5.73 (4.04, 7.42)	4.10 (2.41, 5.79)	4.68 (2.99, 6.37)
	Difference in decrease	2.02 (−0.44, 4.47)	2.81 (0.35, 5.26)	2.90 (0.42, 5.37)	3.95 (1.46, 6.45)
	*p*-value	0.105	0.026 *	0.022 *	0.002 **
IES-R-K-intrusion	HM group	3.12 (2.14, 4.10)	1.86 (0.88, 2.83)	1.20 (0.20, 2.19)	0.58 (−0.43, 1.59)
	Control group	4.33 (3.35, 5.31)	2.65 (1.67, 3.63)	2.54 (1.56, 3.52)	2.91 (1.93, 3.89)
	Difference in decrease	1.21 (−0.21, 2.64)	0.79 (−0.63, 2.22)	1.35 (−0.09, 2.78)	2.33 (0.88, 3.78)
	*p*-value	0.093	0.27	0.066	0.002 **
IES-R-K- sleep problem and numbness	HM group	3.49 (2.60, 4.38)	2.49 (1.60, 3.38)	2.21 (1.30, 3.12)	1.07 (0.14, 2.00)
	Control group	4.26 (3.37, 5.15)	3.32 (2.43, 4.21)	3.42 (2.53, 4.31)	3.42 (2.53, 4.31)
	Difference in decrease	0.78 (−0.51, 2.06)	0.83 (−0.46, 2.11)	1.21 (−0.09, 2.51)	2.35 (1.04, 3.67)
	*p*-value	0.233	0.203	0.067	<0.001 ***
IES-R-K-total	HM group	13.70 (9.84, 17.56)	9.70 (5.84, 13.56)	5.75 (1.83, 9.67)	3.07 (−0.91, 7.04)
	Control group	19.04 (15.17, 22.91)	15.46 (11.59, 19.33)	13.72 (9.85, 17.59)	14.20 (10.33, 18.06)
	Difference in decrease	5.33 (−0.29, 10.96)	5.75 (0.13, 11.38)	7.97 (2.29, 13.64)	11.13 (5.41, 16.84)
	*p*-value	0.063	0.045 *	0.007 **	<0.001 ***
WPAI (work and activity impairment)	HM group	48.57 (42.69, 54.45)	29.44 (23.56, 35.33)	21.62 (15.63, 27.62)	17.41 (11.31, 23.51)
	Control group	61.47 (55.58, 67.37)	50.50 (44.61, 56.39)	46.72 (40.83, 52.61)	38.00 (32.11, 43.89)
	Difference in decrease	12.90 (4.34, 21.47)	21.06 (12.49, 29.62)	25.10 (16.44, 33.75)	20.59 (11.86, 29.32)
	*p*-value	0.004 **	<0.001 ***	<0.001 ***	<0.001 ***
EQ−5D−5L	HM group	0.82 (0.78, 0.85)	0.83 (0.79, 0.86)	0.86 (0.82, 0.89)	0.90 (0.87, 0.94)
	Control group	0.77 (0.74, 0.81)	0.80 (0.76, 0.83)	0.82 (0.79, 0.86)	0.82 (0.78, 0.85)
	Difference in decrease	−0.04 (−0.10, 0.01)	−0.03 (−0.08, 0.02)	−0.03 (−0.09, 0.02)	−0.09 (−0.14, −0.03)
	*p*-value	0.087	0.299	0.203	0.002 **
SF−12 (PCS)	HM group	45.35 (42.57, 48.12)	48.15 (45.37, 50.93)	48.65 (45.82, 51.49)	53.17 (50.27, 56.06)
	Control group	43.92 (41.13, 46.70)	45.77 (42.99, 48.56)	46.89 (44.10, 49.68)	48.96 (46.17, 51.74)
	Difference in decrease	−1.43 (−5.46, 2.60)	−2.38 (−6.40, 1.65)	−1.76 (−5.84, 2.31)	−4.21 (−8.33, −0.09)
	*p*-value	0.481	0.244	0.391	0.045 *
SF−12 (MCS)	HM group	52.77 (49.52, 56.02)	53.04 (49.79, 56.29)	56.25 (52.93, 59.58)	55.87 (52.47, 59.27)
	Control group	48.77 (45.51, 52.03)	51.98 (48.72, 55.25)	52.11 (48.85, 55.37)	51.35 (48.09, 54.61)
	Difference in decrease	−4.00 (−8.70, 0.70)	−1.05 (−5.76, 3.65)	−4.14 (−8.91, 0.62)	−4.52 (−9.33, 0.29)
	*p*-value	0.095	0.657	0.087	0.065
PGIC	HM group	—	1.96 (1.54, 2.39)	1.96 (1.53, 2.39)	1.68 (1.24, 2.11)
	Control group	—	2.51 (2.09, 2.94)	2.30 (1.88, 2.73)	2.15 (1.72, 2.57)
	Difference in decrease	—	−0.55 (−1.17, 0.07)	−0.34 (−0.96, 0.28)	−0.47 (−1.09, 0.16)
	*p*-value	—	0.079	0.275	0.14

The primary analysis was performed with a linear mixed model. The primary endpoint is week 5. All values are presented with least square estimates at a 95% confidence interval. * *p* < 0.05; ** *p* < 0.01; *** *p* < 0.001. HM, herbal medicine; NRS, numeric rating scale; IES-R-K, impact of event scale-revised-Korean; WPAI, work productivity and activities impairment; EQ-5D-5L, EuroQoL 5-dimension 5-level instrument; SF-12, 12-item short-form health survey; PCS, physical component summary; MCS, mental component summary; PGIC, patient global impression of change. * *p* < 0.05, ** *p* < 0.01, *** *p* < 0.001

**Table 4:** Areas under the curves for the 17-week outcomes.

	HM Group	Control Group	Difference	*p*-Value
NRS score for				
Overall post-accident syndromes	45.86 (38.14, 53.58)	69.21 (62.20, 76.23)	−23.35 (−34.36, −12.34)	<0.001 ***
Musculoskeletal complaints	49.11 (40.90, 57.32)	71.35 (63.95, 78.76)	−22.25 (−33.94, −10.55)	<0.001 ***
Neck	41.23 (31.89, 50.57)	58.75 (50.45, 67.05)	−17.52 (−31.17, −3.88)	0.014 *
Lower back	42.37 (32.38, 52.36)	64.43 (54.61, 74.25)	−22.06 (−37.09, −7.02)	0.005 **
Shoulder	36.82 (25.77, 47.86)	63.99 (55.87,72.12)	−27.18 (−42.27, −12.09)	0.001 **
Knee	26.76 (15.29, 38.24)	36.90 (24.47, 49.34)	−10.14 (−29.75, 9.47)	0.285
Neurological complaints	24.48 (12.30, 36.67)	44.74 (33.62, 55.85)	−20.25 (−38.85, −1.66)	0.034 *
Headache	27.01 (13.50, 40.52)	45.81 (33.93, 57.69)	−18.80 (−39.69, 2.08)	0.074
Dizziness	17.83 (−5.44, 41.11)	39.43 (23.06, 55.79)	−21.59 (−57.68, 14.50)	0.207
Psychiatric complaints	32.80 (24.30, 41.31)	58.22 (49.98, 66.46)	−25.42 (−38.50, −12.34)	<0.001 ***
Anxiety	29.36 (18.49, 40.23)	43.56 (33.16, 53.96)	−14.20 (−31.11, 2.71)	0.095
Depression	32.77 (20.30, 45.25)	43.86 (32.11, 55.61)	−11.09 (−30.50, 8.32)	0.239
Anger	23.17 (12.50, 33.83)	45.45 (34.59, 56.30)	−22.28 (−39.91, −4.65)	0.017 *
Insomnia	28.88 (16.65, 41.11)	58.71 (47.88, 69.54)	−29.83 (−48.46, −11.19)	0.003 **
Digestive systemic complaints	14.81 (0.42, 29.20)	26.66 (14.20, 39.12)	−11.85 (−34.72, 11.02)	0.281
Indigestion	16.95 (3.43, 30.47)	29.92 (15.63, 44.21)	−12.97 (−37.08, 11.13)	0.255
Loss of appetite	10.78 (−16.49, 38.04)	25.67 (7.90, 43.43)	−14.89 (−58.72, 28.95)	0.416
General symptoms	40.18 (31.44, 48.93)	67.36 (59.51, 75.21)	−27.17 (−39.80, −14.54)	<0.001 ***
Fatigue	42.23 (32.49, 51.98)	69.11 (60.07, 78.15)	−26.88 (−40.99, −12.76)	<0.001 ***
General weakness	23.68 (14.02, 33.33)	52.26 (41.56, 62.96)	−28.58 (−44.47, −12.69)	0.001 **
IES-R-K-hyperarousal	33.26 (19.14, 47.38)	61.08 (46.95, 75.21)	−27.82 (−49.52, −6.13)	0.014 *
IES-R-K-avoidance	30.13 (11.15, 49.11)	75.93 (56.68, 95.18)	−45.80 (−74.86, −16.74)	0.003 **
IES-R-K-intrusion	31.01 (19.04, 42.98)	49.46 (37.94, 60.98)	−18.45 (−36.51, −0.39)	0.046 *
IES-R-K-sleep problem and numbness	37.29 (27.06, 47.52)	56.93 (46.72, 67.14)	−19.64 (−35.16, −4.11)	0.015 *
IES-R-K-total	131.21 (84.24, 178.18)	243.88 (196.73, 291.03)	−112.67 (−184.63, −40.70)	0.003 **
WPAI	455.06 (376.41, 533.71)	767.49 (693.58, 841.40)	−312.43 (−428.24, −196.62)	<0.001 ***
EQ-5D-5L	13.49 (13.00, 13.98)	12.82 (12.34, 13.30)	0.67 (−0.09 to 1.43)	0.082
SF-12 (PCS)	770.22 (737.12, 803.32)	745.00 (712.05, 777.95)	25.22 (−25.29, 75.72)	0.317
SF-12 (MCS)	868.60 (831.44, 905.76)	817.36 (781.07, 853.65)	51.24 (−4.73, 107.22)	0.071

The area under the curve was calculated using the trapezoidal rule. The differences between the two groups were analyzed using an analysis of covariance. Missing values were imputed using multiple imputations. All values are presented with least square estimates with a 95% confidence interval. * *p* < 0.05; ** *p* < 0.01; *** *p* < 0.001. HM, herbal medicine; NRS, numeric rating scale for symptom used by patients to report their symptom level as a number from 0 (no symptom) to 10 (most severe symptom imaginable); IES-R-K, impact of event scale-revised-Korean with scores calculated by the severity of symptoms on a 5-point scale from 0 (not at all) to 4 (extremely) based on the past week, with an overall total score of 0–88; WPAI, work productivity and activities impairment; EQ-5D-5L, EuroQoL 5-dimension 5-level instrument with scores calculated by converting the patients’ responses to a scale ranging from −0.066 (lowest quality of life) to 1 (highest quality of life); SF-12, 12-item short-form health survey with scores calculated by converting the patients’ responses to a scale ranging from 0 (lowest quality of life) to 100 (highest quality of life); PCS, physical component summary; MCS, mental component summary.

## Data Availability

We plan to share our findings with the participants, healthcare professionals, and the public through the publication of this report or trial registries. Data and materials can be requested by e-mail and will be provided after consultation with the IRB.

## Supplementary Materials

The following supporting information can be downloaded at: https://www.mdpi.com/article/10.3390/healthcare11040534/s1, Table S1: Schedule of the participants; Table S2: List of treatments provided to the patients; Table S3: Types of prescribed herbal medicine and prescription days for the HM group (*n* = 20); Table S4: Sensitivity analysis of between-group differences with multiple imputations; Table S5: Sensitivity analysis of between-group differences with last observation carried forward; Table S6: Analysis of between-group differences with the linear mixed model for subdivided symptoms; Table S7: Prescription history of medication.
