# Supplementary material for: Efficacy and Safety of Korean Herbal Medicine for Patients with Post-Accident Syndrome, Persistent after Acute Phase: A Pragmatic Randomized Controlled Trial
Source: Healthcare (Basel). 2023 Feb 10;11(4):534. doi: 10.3390/healthcare11040534 (PMC9957496; doi:10.3390/healthcare11040534)
Supplement: Supplementary file 1 [file healthcare-11-00534-s001.zip › healthcare-2138156-supplementary.pdf]

Table S1. Schedule of the participants.

Time Point (Week)	Screening	Enrolment Allocation	Active Treatment				Follow-Up		
	–1	0	1	2	3	4	5	9	17
Visit window	–7	Control point	±3	±3	±3	±3	±4	±7	±14
**Enrolment**									
Eligibility screening	○	–	–	–	–	–	–	–	–
Informed consent form	○	–	–	–	–	–	–	–	–
Confirm suitability for study	○	–	–	–	–	–	–	–	–
Vital signs	○	○	← Every visit →				○	○	○
Sociodemographic characteristics	○	–	–	–	–	–	–	–	–
Medical history	○	–	–	–	–	–	–	–	–
Credibility and Expectancy Questionnaire	○	–	–	–	–	–	–	–	–
Block randomization	–	○	–	–	–	–	–	–	–
**Intervention**									
Treatment in the Herbal Medicine group			← 2 (1–3) times/week → (taking herbal medicine)						
Treatment in the Control group			← 2 (1–3) times/week →						
**Assessment**									
Drug consumption	○	○	← Every visit →				○	○	○
NRS of post-accident syndromes	○	–	○	–	○	–	○	○	○
IES-R-K	–	–	○	–	○	–	○	○	○
EQ-5D-5L	–	–	○	–	○	–	○	○	○
SF-12	–	–	○	–	○	–	○	○	○
PGIC	–	–	–	–	–	–	○	○	○
WPAI	–	–	○	○	○	○	○	○	○
Adverse events	–	○	← Every visit →				○	○	○
Blood analysis	○	–	–	–	–	–	○	–	–

NRS, numeric rating scale; IES-R-K, impact of event scale-revised-Korean; EQ-5D-5L, EuroQoL 5-dimension 5-level instrument; SF-12, 12-item short form health survey; PGIC, patient global impression of change; WPAI, work productivity and activity impairment.

Table S2. List of IKM treatments provided to the patients

	**HM Group (*n* = 20)**		**Control Group (*n* = 20)**	
	N (%)	Mean ± SD	N (%)	Mean ± SD
Acupuncture	20 (100.0)	6.60 ± 1.88	20 (100.0)	5.40 ± 1.64
Cupping	20 (100.0)	6.40 ± 2.23	20 (100.0)	5.35 ± 1.76
Pharmacopuncture	19 (95.0)	4.05 ± 1.54	20 (100.0)	4.25 ± 1.02
Interferential current therapy	12 (60.0)	4.05 ± 3.75	13 (65.0)	3.65 ± 2.98
Doin therapy	12 (60.0)	3.70 ± 3.44	8 (40.0)	2.15 ± 2.92
Moxibustion	6 (30.0)	2.25 ± 3.55	8 (40.0)	1.85 ± 2.58
Chuna manual therapy	3 (15.0)	0.45 ± 1.23	10 (50.0)	1.45 ± 1.93

The numbers (%) of patients in both groups who received each treatment and the mean ± SD days of the treatments are shown. Multiple treatment uses were allowed. HM, herbal medicine; IKM, integrative Korean medicine; SD, standard deviation.

Table S3. Types of prescribed herbal medicine and prescription days for the HM group (n = 20)

Intervention	N (%)	Mean ± SD
Yukgong-dan (六拱丹)	12 (60.0)	23.33 ± 6.89
Gwanjeol-go (關節膏)	9 (45.0)	23.33 ± 7.00
Chungpa-Jeon H	8 (40.0)	21.00 ± 7.48
Gamiseogyeong-tang (加味舒經湯)	7 (35.0)	24.50 ± 7.00
Yukgongbaro-hwan	2 (10.0)	14.00 ± 0.00
Chungpajeonsinbang 2	2 (10.0)	14.00 ± 0.00
Gamidokhwalgisaeng-tang (加味獨活寄生湯)	1 (5.0)	28.00 ± NA
Gamisoyo-san (加味逍遙散)	1 (5.0)	28.00 ± NA
Gaegyeolseogyeong-tang (開結舒經湯)	1 (5.0)	28.00 ± NA
Yukgong-dan (六拱丹) premium	1 (5.0)	28.00 ± NA
Yanggeunbaro-hwan	1 (5.0)	28.00 ± NA
Chungpa-Jeon	1 (5.0)	28.00 ± NA
Gamiondam-tang (加味溫膽湯)	1 (5.0)	14.00 ± NA
Madae-hwan (麻大丸)	1 (5.0)	14.00 ± NA
Boikyangwi-tang (補益養胃湯)	1 (5.0)	14.00 ± NA
Sungihwalhyeol-tang (順氣活血湯)	1 (5.0)	14.00 ± NA
Yeongolbogang-hwan (軟骨補強丸)	1 (5.0)	14.00 ± NA
Ungeoonbi-hwan	1 (5.0)	14.00 ± NA
Gongjin-dan (拱辰丹)	1 (5.0)	14.00 ± NA
Cheongsinbaro-hwan	1 (5.0)	14.00 ± NA
Cheongungbaro-hwan	1 (5.0)	14.00 ± NA
Cheongungyeonbo	1 (5.0)	14.00 ± NA
Chungpayanggeun-tang	1 (5.0)	14.00 ± NA
Hyangsoijin-tang (香蘇二陳湯)	1 (5.0)	14.00 ± NA
Bunso-san	1 (5.0)	4.00 ± NA
Sanjoin-tang (酸棗仁湯)	1 (5.0)	2.00 ± NA
Sodojisa-san (消導止瀉散)	1 (5.0)	2.00 ± NA

The number (%) of patients and prescription days are shown. "NA" indicates that the herbal medicine was prescribed for only one participant; thus, the SD could not be obtained. NA, not applicable; SD, standard deviation; HM, herbal medicine.

Table S4. Sensitivity analysis of between-group differences with multiple imputations

		Week 3	Week 5	Week 9	Week 17
**NRS Score for Overall Post-Accident Syndromes**	HM group	4.68 (4.25, 5.12)	2.85 (2.33, 3.36)	2.41 (1.74, 3.09)	1.79 (0.98, 2.60)
	Control group	5.52 (5.08, 5.96)	4.63 (4.11, 5.14)	4.02 (3.44, 4.60)	3.47 (2.87, 4.07)
	Difference in decrease	0.84 (0.17, 1.50)	1.78 (1.01, 2.55)	1.60 (0.66, 2.54)	1.68 (0.62, 2.74)
	p value	0.015*	<0.001***	0.002**	0.003**
**NRS Score for Musculoskeletal Complaints**	HM group	4.84 (4.34, 5.34)	3.11 (2.53, 3.69)	2.49 (1.72, 3.26)	2.08 (1.20, 2.95)
	Control group	5.79 (5.31, 6.28)	4.78 (4.20, 5.36)	4.16 (3.50, 4.83)	3.40 (2.75, 4.04)
	Difference in decrease	0.95 (0.19, 1.70)	1.67 (0.82, 2.52)	1.68 (0.60, 2.75)	1.32 (0.20, 2.44)
	p value	0.016*	<0.001***	0.004**	0.023*
**NRS Score for Neurological Complaints**	HM group	2.99 (2.20, 3.77)	1.88 (0.90, 2.86)	1.11 (–0.03, 2.26)	0.47 (–0.30, 1.24)
	Control group	4.00 (3.30, 4.70)	3.33 (2.44, 4.22)	2.58 (1.56, 3.61)	1.74 (1.05, 2.42)
	Difference in decrease	1.01 (–0.17, 2.19)	1.45 (–0.04, 2.94)	1.47 (–0.28, 3.22)	1.27 (0.10, 2.44)
	p value	0.089	0.055	0.095	0.035*
**NRS Score for Psychiatric Complaints**	HM group	3.91 (3.40, 4.42)	2.53 (1.79, 3.27)	1.44 (0.69, 2.19)	0.85 (0.07, 1.63)
	Control group	4.67 (4.18, 5.15)	4.01 (3.30, 4.72)	3.35 (2.65, 4.06)	2.85 (2.10, 3.60)
	Difference in decrease	0.76 (0.00, 1.52)	1.48 (0.35, 2.60)	1.91 (0.78, 3.05)	1.99 (0.81, 3.18)
	p value	0.051	0.012*	0.002**	0.002**
**NRS Score for Digestive Systemic Complaints**	HM group	2.07 (0.96, 3.19)	0.91 (–0.10, 1.92)	0.49 (–0.59, 1.57)	0.31 (–0.77, 1.38)
	Control group	2.33 (1.41, 3.25)	1.49 (0.63, 2.35)	1.49 (0.57, 2.41)	1.21 (0.28, 2.13)
	Difference in decrease	0.26 (–1.51, 2.02)	0.58 (–1.03, 2.18)	1.00 (–0.71, 2.71)	0.90 (–0.81, 2.61)
	p value	0.756	0.446	0.227	0.274
**NRS Score for General Symptoms**	HM group	4.13 (3.61, 4.66)	2.60 (1.99, 3.20)	2.04 (1.21, 2.88)	1.64 (0.53, 2.75)
	Control group	4.96 (4.40, 5.51)	4.52 (3.91, 5.13)	3.97 (3.27, 4.67)	3.69 (2.89, 4.49)
	Difference in decrease	0.82 (0.01, 1.64)	1.92 (1.05, 2.79)	1.93 (0.79, 3.06)	2.05 (0.61, 3.49)
	p value	0.047*	<0.001***	0.002**	0.008**
**IES-R-K-Hyperarousal**	HM group	3.29 (2.23, 4.35)	2.55 (1.42, 3.69)	1.49 (0.43, 2.54)	0.96 (–0.25, 2.16)
	Control group	5.18 (4.11, 6.24)	3.69 (2.57, 4.80)	3.59 (2.54, 4.63)	2.97 (1.78, 4.16)
	Difference in decrease	1.89 (0.28, 3.50)	1.13 (–0.58, 2.84)	2.10 (0.47, 3.73)	2.02 (0.17, 3.86)
	p value	0.023*	0.188	0.013*	0.034*
**IES-R-K-Avoidance**	HM group	3.21 (1.76, 4.67)	3.06 (1.06, 5.06)	1.10 (–0.13, 2.32)	0.39 (–1.18, 1.97)

	Control group	5.63 (4.11, 7.14)	5.51 (3.46, 7.56)	3.98 (2.77, 5.18)	4.48 (2.89, 6.06)
	Difference in decrease	2.41 (0.15, 4.67)	2.45 (–0.63, 5.53)	2.88 (1.03, 4.73)	4.08 (1.67, 6.49)
	p value	0.037*	0.115	0.003**	0.002**
**IES-R-K-Intrusion**	HM group	2.99 (1.90, 4.08)	1.91 (0.91, 2.92)	1.51 (0.66, 2.37)	1.01 (0.10, 1.92)
	Control group	4.54 (3.47, 5.62)	2.69 (1.70, 3.67)	2.50 (1.67, 3.32)	2.79 (1.96, 3.61)
	Difference in decrease	1.55 (–0.10, 3.20)	0.78 (–0.75, 2.30)	0.98 (–0.30, 2.27)	1.78 (0.41, 3.14)
	p value	0.064	0.307	0.129	0.013*
**IES-R-K-Sleep Problem and Numbness**	HM group	3.23 (2.48, 3.98)	2.44 (1.49, 3.38)	2.35 (1.57, 3.14)	1.14 (0.11, 2.17)
	Control group	4.52 (3.77, 5.26)	3.37 (2.42, 4.32)	3.32 (2.55, 4.08)	3.27 (2.31, 4.23)
	Difference in decrease	1.29 (0.15, 2.42)	0.93 (–0.51, 2.37)	0.96 (–0.21, 2.13)	2.13 (0.61, 3.64)
	p value	0.027*	0.196	0.105	0.007**
**IES-R-K-Total**	HM group	12.68 (9.52, 15.84)	9.92 (5.84, 13.99)	6.42 (3.31, 9.52)	3.49 (–0.37, 7.34)
	Control group	19.91 (16.71, 23.10)	15.29 (11.18, 19.41)	13.41 (10.30, 16.52)	13.52 (9.72, 17.31)
	Difference in decrease	7.23 (2.39, 12.06)	5.38 (–0.88, 11.63)	6.99 (2.24, 11.75)	10.03 (4.17, 15.90)
	p value	0.005**	0.09	0.005**	0.001**
**WPAI (Work and Activity Impairment)**	HM group	48.55 (42.63, 54.48)	29.36 (23.46, 35.27)	21.77 (14.34, 29.20)	18.37 (10.82, 25.92)
	Control group	61.14 (55.69, 66.58)	50.73 (44.91, 56.55)	45.84 (38.91, 52.78)	38.05 (31.80, 44.30)
	Difference in decrease	12.59 (4.06, 21.11)	21.37 (12.32, 30.41)	24.07 (13.22, 34.92)	19.68 (9.26, 30.11)
	p value	0.005**	<0.001***	<0.001***	<0.001***
**EQ-5D-5L**	HM group	0.81 (0.78, 0.83)	0.82 (0.79, 0.85)	0.85 (0.81, 0.89)	0.90 (0.85, 0.95)
	Control group	0.77 (0.74, 0.79)	0.79 (0.76, 0.82)	0.82 (0.78, 0.86)	0.81 (0.77, 0.85)
	Difference in decrease	–0.04 (–0.08, 0.00)	–0.02 (–0.07, 0.02)	–0.03 (–0.09, 0.03)	–0.09 (–0.16, –0.02)
	p value	0.064	0.309	0.355	0.019*
**SF-12 (PCS)**	HM group	45.29 (42.82, 47.77)	48.96 (46.61, 51.32)	47.51 (44.56, 50.46)	51.69 (48.44, 54.94)
	Control group	43.92 (41.43, 46.41)	44.89 (42.52, 47.26)	47.28 (44.44, 50.12)	49.47 (46.48, 52.47)
	Difference in decrease	–1.37 (–5.14, 2.39)	–4.07 (–7.65, –0.50)	–0.23 (–4.67, 4.20)	–2.21 (–7.06, 2.63)
	p value	0.463	0.027*	0.916	0.357
**SF-12 (MCS)**	HM group	52.62 (49.43, 55.82)	52.45 (49.28, 55.62)	55.83 (53.10, 58.55)	55.43 (51.45, 59.40)
	Control group	48.77 (45.54, 52.01)	52.12 (48.96, 55.27)	51.52 (48.90, 54.13)	51.26 (47.40, 55.12)
	Difference in decrease	–3.85 (–8.73, 1.03)	–0.33 (–5.14, 4.47)	–4.31 (–8.41, –10.22)	–4.17 (–10.11, 1.78)
	p value	0.118	0.889	0.040*	0.163

**PGIC**	HM group	–	1.91 (1.59, 2.23)	1.99 (1.54, 2.44)	1.75 (1.30, 2.21)
	Control group	–	2.55 (2.23, 2.87)	2.28 (1.85, 2.72)	2.10 (1.65, 2.54)
	Difference in decrease	–	–0.64 (–1.13, –0.16)	–0.29 (–0.96, 0.38)	–0.34 (–1.03, 0.35)
	p value	–	0.011*	0.381	0.322

The sensitivity analysis was performed with multiple imputation. Each group was compared using analysis of covariance. The primary endpoint is week 5. All values are presented with least square estimates and its 95% confidence interval. *$p < 0.05$; **$p < 0.01$; ***$p < 0.001$. SD, standard deviation; HM, herbal medicine; NRS, numeric rating scale; IES-R-K, impact of event scale-revised-Korean; WPAI, work productivity and activity impairment; EQ-5D-5L, EuroQoL 5-dimension 5-level instrument; SF-12, 12-item short form health survey; PCS, physical component summary; MCS, mental component summary; PGIC, patient global impression of change.

Table S5. Sensitivity analysis of between-group differences with last observation carried forward

		Week 3	Week 5	Week 9	Week 17
**NRS Score for Overall Post-Accident Syndromes**	HM group	4.73 (5.16, 4.31)	2.97 (3.51, 2.42)	2.52 (3.11, 1.92)	1.98 (2.66, 1.31)
	Control group	5.57 (5.99, 5.14)	4.73 (5.28. 4.19)	4.13 (4.73, 3.54)	3.62 (4.29, 2.94)
	Difference in decrease	0.83 (0.21, 1.46)	1.77 (0.96, 2.57)	1.61 (0.74, 2.48)	1.64 (0.65, 2.62)
	p value	0.011*	<0.001***	<0.001***	0.002**
**NRS Score for Musculoskeletal Complaints**	HM group	4.93 (5.42, 4.45)	3.31 (3.92, 2.69)	2.79 (3.53, 2.06)	2.30 (3.07, 1.52)
	Control group	5.87 (6.35, 5.38)	4.94 (5.56, 4.33)	4.31 (5.04, 3.57)	3.60 (4.38, 2.83)
	Difference in decrease	0.93 (0.22, 1.65)	1.64 (0.73, 2.55)	1.51 (0.44, 2.59)	1.31 (0.17, 2.44)
	p value	0.012*	<0.001***	0.007**	0.026*
**NRS Score for Neurological Complaints**	HM group	3.00 (3.76, 2.25)	1.90 (2.87, 0.93)	1.20 (2.34, 0.07)	0.63 (1.39, –0.12)
	Control group	4.00 (4.72, 3.27)	3.32 (4.25, 2.39)	2.58 (3.67, 1.49)	1.72 (2.44, 1.00)
	Difference in decrease	0.99 (–0.10, 2.09)	1.43 (0.02, 2.83)	1.38 (–0.27, 3.03)	1.09 (–0.01, 2.18)
	p value	0.073	0.048*	0.096	0.051
**NRS Score for Psychiatric Complaints**	HM group	3.96 (4.44, 3.48)	2.59 (3.31, 1.87)	1.63 (2.35, 0.90)	1.19 (1.98, 0.39)
	Control group	4.69 (5.17, 4.20)	4.06 (4.77, 3.34)	3.37 (4.10, 2.65)	2.87 (3.66, 2.08)
	Difference in decrease	0.72 (0.01, 1.44)	1.47 (0.40, 2.53)	1.75 (0.68, 2.82)	1.68 (0.51, 2.86)
	p value	0.046*	0.008**	0.002**	0.007**
**NRS Score for Digestive Systemic Complaints**	HM group	2.26 (3.38, 1.14)	1.05 (2.14, –0.04)	0.69 (1.87, –0.49)	0.55 (1.75, –0.64)
	Control group	2.29 (3.27, 1.31)	1.46 (2.42, 0.50)	1.45 (2.48, 0.41)	1.16 (2.21, 0.11)
	Difference in decrease	0.03 (–1.57, 1.64)	0.41 (–1.16, 1.97)	0.76 (–0.94, 2.45)	0.60 (–1.12, 2.32)
	p value	0.967	0.587	0.356	0.464
**NRS Score for General Symptoms**	HM group	4.11 (4.54, 3.68)	2.63 (3.14, 2.12)	2.07 (2.65, 1.50)	1.50 (2.14, 0.86)
	Control group	5.00 (5.43, 4.57)	4.54 (5.05, 4.03)	4.04 (4.61, 3.46)	3.72 (4.37, 3.08)
	Difference in decrease	0.89 (0.25, 1.52)	1.91 (1.16, 2.67)	1.96 (1.12, 2.81)	2.22 (1.27, 3.17)
	p value	0.008**	<0.001***	<0.001***	<0.001***
**IES-R-K-Hyperarousal**	HM group	3.32 (4.38, 2.26)	2.58 (3.70, 1.47)	1.67 (2.76, 0.57)	1.39 (2.66, 0.12)
	Control group	5.33 (6.39, 4.27)	3.87 (4.98, 2.75)	3.68 (4.78, 2.59)	3.01 (4.28, 1.74)
	Difference in decrease	2.01 (0.46, 3.57)	1.28 (–0.36, 2.93)	2.02 (0.40, 3.64)	1.62 (–0.25, 3.49)
	p value	0.013*	0.123	0.016*	0.088
**IES-R-K-Avoidance**	HM group	3.29 (4.75, 1.84)	3.07 (5.07, 1.06)	1.71 (3.26, 0.16)	1.26 (3.16, –0.63)

	Control group	5.86 (7.31, 4.40)	5.73 (7.74, 3.73)	4.19 (5.74, 2.64)	4.64 (6.53, 2.74)
	Difference in decrease	2.56 (0.42, 4.70)	2.67 (–0.27, 5.61)	2.49 (0.21, 4.76)	3.38 (0.59, 6.16)
	p value	0.020*	0.074	0.033*	0.019*
**IES-R-K-Intrusion**	HM group	3.04 (4.13, 1.96)	2.05 (3.08, 1.02)	1.55 (2.39, 0.71)	1.09 (1.94, 0.24)
	Control group	4.61 (5.69, 3.52)	2.80 (3.83, 1.77)	2.60 (3.44, 1.76)	2.86 (3.71, 2.01)
	Difference in decrease	1.56 (–0.03, 3.16)	0.75 (–0.76, 2.26)	1.05 (–0.19, 2.29)	1.77 (0.51, 3.02)
	p value	0.055	0.322	0.093	0.007**
**IES-R-K-Sleep Problem and Numbness**	HM group	3.34 (4.09, 2.58)	2.61 (3.58, 1.64)	2.58 (3.38, 1.79)	1.65 (2.67, 0.62)
	Control group	4.46 (5.22, 3.71)	3.34 (4.31, 2.37)	3.27 (4.06, 2.47)	3.25 (4.28, 2.23)
	Difference in decrease	1.13 (0.01, 2.24)	0.73 (–0.71, 2.16)	0.69 (–0.48, 1.85)	1.60 (0.10, 3.11)
	p value	0.048*	0.311	0.242	0.038*
**IES-R-K-Total**	HM group	12.96 (16.20, 9.73)	10.29 (14.50, 6.07)	7.50 (11.06, 3.94)	5.41 (9.74, 1.08)
	Control group	20.29 (23.52, 17.05)	15.76 (19.98, 11.55)	13.75 (17.31, 10.19)	13.74 (18.07, 9.41)
	Difference in decrease	7.32 (2.56, 12.09)	5.47 (–0.73, 11.68)	6.25 (1.01, 11.50)	8.33 (1.96, 14.70)
	p value	0.004**	0.082	0.021*	0.012*
**WPAI (Work and Activity Impairment)**	HM group	49.57 (54.89, 44.25)	31.12 (37.46, 24.79)	25.63 (33.31, 17.96)	21.01 (28.68, 13.35)
	Control group	61.49 (66.81, 56.17)	51.34 (57.68, 45.00)	46.90 (54.58, 39.23)	39.04 (46.70, 31.37)
	Difference in decrease	11.92 (4.06, 19.78)	20.22 (10.85, 29.58)	21.27 (9.94, 32.61)	18.02 (6.70, 29.35)
	p value	0.004**	<0.001***	<0.001***	0.003**
**EQ-5D-5L**	HM group	0.81 (0.84, 0.79)	0.82 (0.85, 0.79)	0.85 (0.89, 0.81)	0.89 (0.93, 0.85)
	Control group	0.77 (0.80, 0.75)	0.79 (0.83, 0.76)	0.82 (0.86, 0.78)	0.81 (0.86, 0.77)
	Difference in decrease	–0.04 (–0.07, 0.00)	–0.03 (–0.07, 0.02)	–0.03 (–0.09, 0.03)	–0.07 (–0.14, –0.01)
	p value	0.034*	0.275	0.298	0.024*
**SF-12 (PCS)**	HM group	45.10 (47.56, 42.65)	48.60 (50.99, 46.20)	47.82 (50.62, 45.02)	51.48 (54.52, 48.44)
	Control group	43.73 (46.18, 41.27)	44.65 (47.05, 42.26)	47.02 (49.82, 44.22)	49.07 (52.11, 46.03)
	Difference in decrease	–1.38 (–4.99, 2.23)	–3.94 (–7.46, –0.42)	–0.80 (–4.92, 3.32)	–2.41 (–6.88, 2.05)
	p value	0.444	0.029*	0.696	0.28
**SF-12 (MCS)**	HM group	52.77 (56.02, 49.52)	52.76 (55.94, 49.58)	55.76 (58.38, 53.14)	55.26 (59.09, 51.43)
	Control group	48.80 (52.05, 45.54)	52.11 (55.29, 48.93)	51.70 (54.32, 49.08)	51.50 (55.33, 47.67)
	Difference in decrease	–3.97 (–8.74, 0.80)	–0.65 (–5.32, 4.02)	–4.07 (–7.91, –0.22)	–3.76 (–9.38, 1.85)
	p value	0.1	0.778	0.039*	0.183

**PGIC**	HM group	–	1.91 (2.25, 1.58)	1.97 (2.43, 1.51)	1.75 (2.22, 1.28)
	Control group	–	2.56 (2.90, 2.23)	2.29 (2.75, 1.84)	2.09 (2.57, 1.62)
	Difference in decrease	–	–0.65 (–1.14, –0.16)	–0.32 (–0.99, 0.35)	–0.35 (–1.04, 0.34)
	p value	–	0.011*	0.332	0.315

The sensitivity analysis was performed with last observation carried forward. Each group was compared using analysis of covariance. The primary endpoint is week 5. All values are presented with least square estimates and its 95% confidence interval. *$p < 0.05$; **$p < 0.01$; ***$p < 0.001$. SD, standard deviation; HM, herbal medicine; NRS, numeric rating scale; IES-R-K, impact of event scale-revised-Korean; WPAI, work productivity and activity impairment; EQ-5D-5L, EuroQoL 5-dimension 5-level instrument; SF-12, 12-item short form health survey; PCS, physical component summary; MCS, mental component summary; PGIC, patient global impression of change.

Table S6. Analysis of between-group differences with the linear mixed model for subdivided symptoms

		Baseline		Week 3	Week 5	Week 9	Week 17
		n (%)	Mean ± SD				
**NRS Score for Musculoskeletal Complaints**							
Neck	HM group	18 (90.0)	6.2 ± 1.4	3.70 (3.07, 4.34)	2.59 (1.95, 3.22)	1.94 (1.29, 2.58)	1.39 (0.73, 2.04)
	Control group	19 (95.0)	5.9 ± 1.3	5.10 (4.50, 5.70)	4.21 (3.61, 4.81)	3.26 (2.66, 3.86)	2.63 (2.03, 3.23)
	Difference in decrease	–	–	1.40 (0.50, 2.30)	1.62 (0.72, 2.52)	1.32 (0.41, 2.23)	1.24 (0.33, 2.16)
	p value	1.000	0.456	0.003**	<0.001***	0.005**	0.009**
Low back	HM group	20 (100.0)	6.0 ± 1.4	4.27 (3.58, 4.96)	2.79 (2.10, 3.48)	2.38 (1.68, 3.08)	1.73 (1.03, 2.44)
	Control group	19 (95.0)	6.4 ± 1.3	5.16 (4.45, 5.88)	4.33 (3.62, 5.04)	3.77 (3.06, 4.49)	3.33 (2.62, 4.04)
	Difference in decrease	–	–	0.90 (–0.13, 1.92)	1.54 (0.51, 2.56)	1.40 (0.37, 2.42)	1.60 (0.56, 2.63)
	p value	1.000	0.466	0.084	0.004**	0.009**	0.003**
Shoulder	HM group	11 (55.0)	4.9 ± 2.3	3.85 (3.00, 4.71)	2.55 (1.70, 3.41)	1.80 (0.91, 2.69)	1.13 (0.25, 2.02)
	Control group	16 (80.0)	5.2 ± 1.7	5.23 (4.55, 5.90)	4.60 (3.93, 5.27)	3.66 (2.99, 4.33)	2.98 (2.30, 3.65)
	Difference in decrease	–	–	1.37 (0.27, 2.48)	2.05 (0.94, 3.15)	1.86 (0.73, 3.00)	1.84 (0.71, 2.98)
	p value	0.176	0.684	0.016*	<0.001***	0.002**	0.002**
Knee	HM group	11 (55.0)	5.4 ± 2.1	3.81 (2.87, 4.76)	1.91 (0.97, 2.86)	1.04 (0.08, 2.00)	0.45 (–0.53, 1.44)
	Control group	9 (45.0)	3.7 ± 1.6	3.11 (2.12, 4.10)	2.44 (1.45, 3.43)	2.33 (1.34, 3.32)	1.44 (0.45, 2.43)
	Difference in decrease	–	–	–0.70 (–2.14, 0.73)	0.53 (–0.91, 1.97)	1.29 (–0.16, 2.74)	0.99 (–0.47, 2.45)
	p value	0.752	0.055	0.322	0.455	0.079	0.176
**NRS Score for Neurological Complaints**							
Headache	HM group	10 (50.0)	4.8 ± 2.3	3.15 (2.10, 4.20)	1.93 (0.88, 2.98)	0.94 (–0.16, 2.05)	0.05 (–1.11, 1.22)
	Control group	11 (55.0)	4.5 ± 1.4	4.29 (3.35, 5.24)	3.20 (2.26, 4.15)	2.75 (1.80, 3.70)	1.84 (0.89, 2.79)
	Difference in decrease	–	–	1.14 (–0.32, 2.61)	1.27 (–0.19, 2.74)	1.81 (0.30, 3.32)	1.79 (0.23, 3.34)
	p value	1.000	0.689	0.123	0.087	0.020*	0.025*
Dizziness	HM group	5 (25.0)	4.0 ± 1.9	2.49 (0.75, 4.23)	1.29 (–0.45, 3.03)	0.69 (–1.05, 2.43)	0.49 (–1.25, 2.23)
	Control group	9 (45.0)	4.0 ± 0.5	3.39 (2.16, 4.62)	2.84 (1.61, 4.07)	2.17 (0.94, 3.40)	1.62 (0.38, 2.85)
	Difference in decrease	–	–	0.90 (–1.40, 3.20)	1.55 (–0.75, 3.85)	1.48 (–0.82, 3.78)	1.12 (–1.18, 3.42)
	p value	0.320	1.000	0.414	0.171	0.189	0.311
**NRS Score for Psychiatric Complaints**							
Anxiety	HM group	14 (70.0)	5.8 ± 1.6	3.81 (3.00, 4.63)	1.97 (1.16, 2.78)	0.93 (0.10, 1.76)	0.70 (–0.14, 1.54)
	Control group	14 (70.0)	4.6 ± 2.2	4.26 (3.45, 5.07)	3.11 (2.29, 3.92)	2.26 (1.45, 3.07)	1.64 (0.83, 2.46)
	Difference in decrease	–	–	0.44 (–0.75, 1.64)	1.14 (–0.06, 2.33)	1.33 (0.12, 2.54)	0.95 (–0.27, 2.17)

	p value	1.000	0.132	0.456	0.062	0.032*	0.125
Depression	HM group	9 (45.0)	5.1 ± 1.5	4.17 (3.16, 5.17)	2.39 (1.39, 3.39)	1.61 (0.61, 2.61)	0.81 (−0.23, 1.85)
	Control group	10 (50.0)	4.7 ± 1.9	3.97 (3.02, 4.92)	3.07 (2.12, 4.02)	2.57 (1.62, 3.52)	1.67 (0.72, 2.62)
	Difference in decrease	–	–	−0.19 (−1.60, 1.22)	0.69 (−0.72, 2.09)	0.96 (−0.45, 2.37)	0.86 (−0.57, 2.30)
	p value	1.000	0.607	0.783	0.329	0.173	0.231
Anger	HM group	10 (50.0)	4.8 ± 2.0	2.55 (1.64, 3.46)	1.45 (0.54, 2.36)	1.05 (0.14, 1.96)	0.74 (−0.21, 1.69)
	Control group	10 (50.0)	4.4 ± 2.2	3.66 (2.69, 4.62)	3.44 (2.47, 4.40)	2.88 (1.92, 3.84)	1.77 (0.81, 2.73)
	Difference in decrease	–	–	1.11 (−0.26, 2.48)	1.99 (0.62, 3.36)	1.83 (0.46, 3.20)	1.03 (−0.37, 2.43)
	p value	1.000	0.680	0.11	0.006**	0.010*	0.146
Insomnia	HM group	12 (60.0)	5.5 ± 2.5	3.71 (2.69, 4.73)	2.26 (1.23, 3.28)	0.98 (−0.04, 2.01)	0.36 (−0.70, 1.42)
	Control group	14 (70.0)	5.1 ± 1.6	5.24 (4.34, 6.14)	4.31 (3.41, 5.21)	3.17 (2.27, 4.06)	2.60 (1.70, 3.49)
	Difference in decrease	–	–	1.53 (0.11, 2.95)	2.05 (0.64, 3.47)	2.18 (0.77, 3.60)	2.24 (0.79, 3.68)
	p value	0.741	0.619	0.035*	0.005**	0.003**	0.003**
**NRS Score for Digestive Systemic Complaints**							
Indigestion	HM group	8 (40.0)	4.6 ± 2.4	2.61 (1.39, 3.84)	0.90 (−0.32, 2.12)	0.19 (−1.04,1.41)	−0.16 (−1.44, 1.11)
	Control group	7 (35.0)	3.3 ± 1.7	2.51 (1.27, 3.74)	1.37 (0.13, 2.60)	1.79 (0.56, 3.03)	1.51 (0.27, 2.74)
	Difference in decrease	–	–	−0.11 (−1.96, 1.75)	0.47 (−1.39, 2.32)	1.61 (−0.25, 3.47)	1.67 (−0.23, 3.57)
	p value	1.000	0.230	0.905	0.604	0.085	0.081
Loss of appetite	HM group	3 (15.0)	3.3 ±1.5	0.50 (−1.73, 2.73)	0.50 (−1.73, 2.73)	0.50 (−1.73, 2.73)	0.50 (−1.73, 2.73)
	Control group	7 (35.0)	3.4 ± 2.4	1.93 (0.48, 3.38)	1.50 (0.05, 2.95)	1.50 (0.05, 2.95)	1.22 (−0.24, 2.67)
	Difference in decrease	–	–	1.43 (−1.24, 4.11)	1.00 (−1.67, 3.68)	1.00 (−1.67, 3.68)	0.72 (−1.96, 3.39)
	p value	0.273	0.942	0.243	0.401	0.401	0.543
**NRS Score for General Symptoms**							
Fatigue	HM group	18 (90.0)	5.0 ± 1.5	4.16 (3.56, 4.76)	2.63 (2.03, 3.23)	1.87 (1.26, 2.48)	1.16 (0.54, 1.77)
	Control group	18 (90.0)	5.6 ± 1.8	4.92 (4.31, 5.52)	4.51 (3.90, 5.11)	4.21 (3.61, 4.82)	3.74 (3.14, 4.35)
	Difference in decrease	–	–	0.76 (−0.12, 1.64)	1.88 (0.99, 2.76)	2.34 (1.45, 3.23)	2.59 (1.69, 3.48)
	p value	1.000	0.325	0.09	<0.001***	<0.001***	<0.001***
General weakness	HM group	16 (80.0)	4.6 ± 2.0	3.21 (2.46, 3.96)	1.54 (0.79, 2.29)	0.74 (−0.01, 1.49)	0.70 (−0.07, 1.46)
	Control group	13 (65.0)	5.2 ± 1.9	4.87 (4.05, 5.68)	3.64 (2.82, 4.45)	2.79 (1.98, 3.60)	2.17 (1.36, 2.99)
	Difference in decrease	–	–	1.66 (0.51, 2.80)	2.09 (0.95, 3.24)	2.05 (0.90, 3.19)	1.48 (0.32, 2.63)
	p value	0.480	0.369	0.005**	<0.001***	<0.001***	0.013*

A between-group comparison was performed for the changes in the NRS of patients with each symptom. The number of patients with the applicable symptom at baseline for each group was presented as number (%), and the value of NRS was presented as mean ± SD. The change from baseline was compared with linear mixed model. The primary

endpoint is week 5. All values are presented with least square estimates and its 95% confidence interval. *$p < 0.05$; **$p < 0.01$; ***$p < 0.001$. CI, confidence interval; SD, standard deviation; HM, herbal medicine; NRS, numeric rating scale.

Table S7. Prescription history of medication

	HM Group (*n* = 20)		Control Group (*n* = 20)	
	Number of prescribed patients	Total prescribed days per patient	Number of prescribed patients	Total prescribed days per patient
Total	2 (10.0)	1.0 ± 0.0	7 (35.0)	7.6 ± 6.2
Active treatment period	1 (5.0)	1.0 ± NA	4 (20.0)	2.0 ± 1.4
NSAIDs				
Aceclofenac	0 (0.0)	–	1 (5.0)	4.0 ± NA
Naproxen	0 (0.0)	–	1 (5.0)	1.0 ± NA
Skeletal muscle relaxants				
Eperisone	0 (0.0)	–	1 (5.0)	4.0 ± NA
H2 receptor blockers				
Cimetidine	0 (0.0)	–	1 (5.0)	4.0 ± NA
GI tract regulators and antispasmodics				
Propulsives	0 (0.0)	–	1 (5.0)	4.0 ± NA
Other analgesics and antipyretics				
Paracetamol (acetaminophen)	0 (0.0)	–	2 (10.0)	1.5 ± 0.7
Tramadol and paracetamol	0 (0.0)	–	1 (5.0)	4.0 ± NA
Unknown	1 (5.0)	1.0 ± NA	0 (0.0)	–
Follow-up period	1 (5.0)	1.0 ± NA	4 (20.0)	11.2 ± 5.6
NSAIDs				
Ibuprofen	1 (5.0)	1.0 ± NA	0 (0.0)	–
Naproxen	0 (0.0)	–	1 (5.0)	4.0 ± NA
Propionic acid derivatives	0 (0.0)	–	2 (10.0)	6.0 ± 1.4
Skeletal muscle relaxants				
Eperisone	0 (0.0)	–	2 (10.0)	6.0 ± 1.4
Hypnotics and sedatives, minor tranquilizer				
Diazepam	0 (0.0)	–	1 (5.0)	13.0 ± NA
GI tract regulators and antispasmodics				
Mosapride	0 (0.0)	–	1 (5.0)	7.0 ± NA
Other antiulcerants				
Other drugs for acid-related disorders	0 (0.0)	–	2 (10.0)	6.0 ± 1.4
Other analgesics and antipyretics				
Paracetamol (acetaminophen)	0 (0.0)	–	1 (5.0)	13.0 ± NA
Paracetamol (acetaminophen), combinations excluding psycholeptics	0 (0.0)	–	1 (5.0)	1.0 ± NA
Tramadol and paracetamol	0 (0.0)	–	1 (5.0)	7.0 ± NA
Unknown	0 (0.0)	–	1 (5.0)	2.0 ± NA

For each type of analgesic, the number (percentage) of patients prescribed that analgesic, and the mean ± SD days of the prescription per person, are displayed. “NA” indicates that the drug was only prescribed once; thus, the SD could not be obtained. NA, not applicable; SD, standard deviation; HM, herbal medicine.
